# Smart room occupancy detection using neural networks and the puma optimization algorithm

**DOI:** 10.1038/s41598-025-29938-8

**Published:** 2025-12-20

**Authors:** El-Sayed M. El-Kenawy, Ahmed Mohamed Zaki, Ebrahim A. Mattar, Amel Ali Alhussan, Doaa Sami Khafaga, Marwa M. Eid, Mervat El-Seddek

**Affiliations:** 1 Department of Communications and Electronics, Delta Higher Institute of Engineering and Technology, Mansoura, 35111 Egypt; 2 Department of Electronics and Communications Engineering, Faculty of Engineering, Mansoura University, Mansoura, 35516 Egypt; 3 Faculty of Artificial Intelligence, Delta University for Science and Technology, Mansoura, 11152 Egypt; 4 Department of Electronics and Communications Engineering, Horus University, New Damietta, 34517 Egypt; 5 Applied Science Research Center, Applied Science Private University, Amman, Jordan; 6 Jadara Research Center, Jadara University, Irbid, 21110 Jordan; 7 Department of Computer Sciences, College of Computer and Information Sciences, Princess Nourah bint Abdulrahman University, P.O. Box 84428, 11671 Riyadh, Saudi Arabia; 8 College of Engineering, University of Bahrain, Isa Town, Bahrain

**Keywords:** Room Occupancy Detection, Neural Networks, Machine Learning, Metaheuristic Optimization, Puma Optimizer, Sine Cosine Optimizer, Smart Buildings, Energy Efficiency, Energy science and technology, Engineering, Mathematics and computing

## Abstract

Room occupancy detection with reasonable accuracy is indispensable for developing innovative building systems that provide energy-efficient management, increased security, and greater comfort. The existing occupancy detection solutions based on traditional sensors suffer from high installation costs, a lack of scalability, and the inability to adapt to dynamic environments. This study proposes an optimized machine learning (ML) approach using a Neural Network (NN) model tailored with a Puma Optimizer Sine Cosine Optimizer (POSC) metaheuristic optimization technique to address these challenges. Based on environmental sensor data, such as temperature, humidity, light intensity, and $\hbox {CO}_2$ levels, the proposed model achieves high accuracy in predicting room occupancy. The optimization process helps reinforce the training of the NN model through a dynamic equilibrium between exploration and exploitation, achieving faster convergence speed and better classification. The model is evaluated and compared on a publicly available dataset with other optimization techniques such as the Genetic Algorithm (GA) and Grey Wolf Optimization (GWO). Experimental results prove that the POSC-optimized NN model achieves superior classification and significantly outperforms conventional ML methods in terms of accuracy, precision, recall, and F1-score. These findings suggest that the combined use of metaheuristic optimization and deep learning can be a practical approach for real-world applications in intelligent building automation. The solutions proposed in this research may contribute to the growing field of intelligent occupancy detection and energy-efficient systems for future smart environments.

## Introduction

The difficulty of predicting how many people would be occupying a given room has emerged as a monumental problem given the current demand for proper utilization and security of resources. Conventional approaches are known to use physical proximity sensors to identify the presence of people and, despite providing reasonable performance, have certain drawbacks^1^. They have high installation and maintenance costs, problems of scalability, and limited flexibility to changes in the environment. For example, systems that incorporate sensors will have issues where there are multiple occupancy signals and where changes in environmental conditions take place^2^. These drawbacks speak to the need for other solution methods that can yield sound and scalable solutions. The ability to predict room occupancy can bring great advantages, including the efficient control of energy use, better physical security for rooms or buildings, and increased levels of comfort for residents or workers in smart homes or offices, respectively^3^. Solving this issue requires integrating techniques of data science and computational science as a way of adopting the best approaches to present complex solutions.

Artificial Intelligence (AI) and Machine Learning (ML) propose completely new ways of tackling rigorous problems and exhibit high performance regarding the analysis of data and the making of predictions^4^. Occupancy detection is a smart application where ML algorithms can easily extract complex patterns and even relations in the datasets. These methods eliminate the mere rule-based system, where systems lack the ability to learn and be adaptable to different environments and situations^5^. Among the AI subsections, Neural Networks (NN) may be used in the given type of classification convincingly. The fact that they are able to approximate non-linear relationships and also to handle data of high dimensionality makes them perfectly suitable for occupancy prediction^6^. A common characteristic of all the ML models is the fact that their good results are a consequence of accurate training and parameter tuning. Standard estimation techniques, as captured by the traditional training methodologies, portray acceptable results as incorporated in the modern optimization strategies that can improve their predictive qualities^7^.

Data analysis serves as the foundation of all procedural analytical activities connected with the application of predictive modeling. The room occupancy dataset is informative, with features such as temperature, humidity, light, and $\hbox {CO}_2$ presence level defining occupancy^8^. Preprocessing forms the initial steps in the utilization of this data and involves cleaning, normalization, as well as balancing the dataset. For instance, normalization is applied to make sure that all features are useful in its learning process, while data balancing minimizes cases where some classes dominate the set^9^. Furthermore, it is a process of feature engineering, which describes the way feature extraction can help to discover other relationships to some extent by creating new features from raw features. By selecting the right data inputs, processing them, and preparing data to be fed to machines, models are ready to address the high variability of real-world data and provide reliable predictions^10^.

Optimization is one of the key drivers needed when it comes to enhancing the performance of ML models, most notably NN^11^. Active learning and soft computing paradigms based on metaheuristic optimization algorithms of swarm intelligence, genetic algorithms, and predator-prey techniques are advantageous in improving model efficiency. Puma Optimizer (PO)^12^, Grey Wolf Optimization (GWO)^13,14^, and Genetic Algorithms (GA) are very effective in producing the best configurations for a model in complex solution spaces^15^.

This paper proposes Puma Optimizer (PO)-Sine Cosine Optimizer (POSC), a hybrid optimization approach tailored to improve NN optimization for the task of occupancy prediction. Thus, POSC helps not just reduce time to train a new model but also enhances how the trained model will perform across various scenarios where swarm intelligence and enhancements for classification objectives are of the essence^16^. In essence, this integration of optimization and ML should be seen as a leap forward in overcoming the shortcomings of training by standard methods.

The main objectives of the paper are:

- To demonstrate the effectiveness of NN in addressing the complexities of room occupancy prediction.
- To develop and implement the POSC optimization technique for improving the performance of NN in classification tasks.
- To conduct a comparative analysis of the POSC-optimized Neural Network against other optimization methods, such as GA and GWO, using key performance metrics.
- To evaluate the practical applicability and scalability of the POSC-optimized NN in real-world scenarios.

The inspiration for this research stems from recognizing that individual metaheuristic algorithms face inherent limitations. While the Puma Optimizer demonstrates strong exploration through adaptive hunting strategies, it struggles with solution refinement during exploitation. Conversely, the Sine Cosine Optimizer excels at exploitation through sinusoidal motion but risks premature convergence. Traditional algorithms like Genetic Algorithms and Grey Wolf Optimization similarly exhibit imbalanced exploration-exploitation trade-offs. This motivated the development of the hybrid POSC framework, which combines the complementary strengths of both algorithms. The synergistic integration enables POSC to maintain population diversity, avoid local optima, and achieve faster convergence with higher classification accuracy for room occupancy detection.

The remainder of this paper is organized as follows. Section Background and related work reviews relevant literature on occupancy prediction and optimization methods. Section Materials and methods describes the dataset, neural network architecture, and the POSC optimization technique. Section Experimental results presents experimental results comparing the performance of various ML models and optimization approaches. Section Discussion provides a comprehensive discussion of the results, highlighting the superior performance and practical implications of the POSC-optimized framework. Finally, Section Conclusion and future directions summarizes key findings and conclusions.

## Background and related work

Occupants are key drivers of energy consumption in buildings, and non-intrusive IoT technology can optimize energy performance while protecting privacy. As demonstrated by^17^, this study evaluated various indoor environmental data for occupancy detection in office rooms using IoT sensors. Data on $\hbox {CO}_2$, temperature, humidity, air quality, sound pressure, and illuminance were collected from two offices in Munich, Germany. ML models, including Random Forest, XGBoost, and dense NN, were trained, and feature importance was analyzed with post-hoc explainability testing. Incorporating key features into a 15-minute sliding window improved temporal dependency modeling. Comparing Random and Bayesian optimization techniques, the study found that six days of sound pressure, $\hbox {CO}_2$, and illuminance data yielded occupancy detection accuracy and F1-scores above 0.95 and 0.93, respectively.

Recognizing the location and activity intensity of indoor occupants is vital for intelligent home systems. As reported by^18^, PIR sensors and ML were used to develop models for detecting zonal locations and activity levels. A 15-node PIR sensor array monitored human and cat behavior over 71 days, revealing distinct signal patterns based on location and activity. Using these features, models built with six algorithms identified SVM as the most effective, achieving 99.7% accuracy for training data and 90.9% for test data. The SVM model also distinguished between human activity and cats with greater than 90% accuracy. At least nine sensors were needed for reliable detection, showcasing the potential of PIR sensors in smart homes.

Advancements on the Internet of Things and ML have enabled large-scale sensor deployment for monitoring environments and predicting thermal comfort. As demonstrated by^19^, traditional non-invasive occupancy detection methods often suffer from poor efficiency due to low-quality datasets and suboptimal ML choices. This study integrates data from cameras and environmental sensors with interactive learning and a rule-based classifier to improve dataset quality and preprocessing. A comprehensive public dataset of over 40,000 records−the largest to date−was created for building occupancy prediction. Using multimodal inputs for regression modeling, the approach surpassed statistical methods in accuracy. Tested in a living room prototype, the model achieved 99.7% accuracy for detecting presence and 99.35% for counting occupants, highlighting the robustness of this method.

Machine Learning is a key tool for developing innovative and high-performance buildings, particularly for applications like occupancy prediction. As outlined in the research by^20^, most studies focus on evaluating ML models’ feasibility and performance, but few address practical applications or scalability. This study proposes a transfer learning approach to overcome challenges such as scaling models across buildings, minimizing training data collection, and ensuring robustness under changing conditions. A deep learning model for room occupancy prediction using indoor climate IoT sensors demonstrated that ground truth data collection could be reduced to two days without compromising performance if normalization techniques were applied. While occupancy level prediction showed slightly lower accuracy, the methodology proved robust and practical. Additionally, the study emphasized presenting performance metrics in user-friendly formats to enhance the market adoption of ML solutions in building environments.

Building occupancy information is critical for energy management, enabling strategies that optimize energy consumption while maintaining occupant comfort. As reported by^21^, this study investigates advanced occupancy modeling techniques for residential buildings using data-driven approaches. Various ML models, including Random Forest, Bayesian Network, Decision Trees, and Support Vector Machines, were evaluated for predicting and classifying occupancy. Using IoT sensors and survey data from a living lab, models were assessed based on accuracy and F1 scores, achieving rates between 70% and 95.96%. Random Forest excelled in capturing occupancy trends, while the Bayesian Network, augmented with expert knowledge, provided detailed zone-specific predictions. The study also addressed privacy concerns in data collection, providing effective strategies for occupancy modeling to improve residential energy efficiency.

Occupancy detection at the room level is critical for enhancing occupant-centric controls in residential buildings. As outlined in the research by^22^, two modeling approaches were assessed for this purpose: a global approach for all rooms and a room-specific approach. Using the XGBoost method, models were trained on a rich dataset containing indoor environmental quality variables and occupancy ground truth data. Features such as time of day, $\hbox {CO}_2$ concentration, and short-term environmental dynamics were identified as the most influential. While both models performed well, especially in predicting bedroom occupancy, their generalizability was tested on data from another residential building. The models maintained strong accuracy for bedrooms but struggled with office spaces due to differences in ventilation patterns and air infiltration. Despite these challenges, XGBoost-based occupancy detection demonstrates robust potential for occupant-aware building performance assessments, with the study’s dataset available as open access.

The application of live occupancy detection can significantly enhance demand-controlled ventilation systems, optimizing indoor air quality and energy performance. As reported in the study by^23^, faster region-based convolutional NN models were trained to detect both the number of occupants and their activities, then deployed on an AI-powered camera for real-time monitoring. Experimental tests in a case study room revealed that the people-counting model achieved an average detection accuracy of 98.9%, outperforming the activity detection model, which had an accuracy of 88.5%. Occupancy profiles were generated based on real-time data about the number of people and their activities. Scenario-based building energy simulations showed that this approach can improve ventilation control by dynamically adjusting based on actual occupancy, addressing the inefficiencies of static ventilation profiles, and enhancing indoor air quality.

Accurately measuring indoor occupancy is crucial for reducing energy use and predicting infection risks. As noted by^24^, this study used ML to estimate occupancy based on carbon dioxide levels, differential pressure, and ventilation system status, accounting for fluctuations caused by variables like open doors and windows. Data from a living lab were used to train models, with the Artificial NN achieving the lowest root mean squared error when combining $\hbox {CO}_2$ concentration, differential pressure, and ventilation data. Future work will aim to improve accuracy by incorporating more Internet of Things sensors and expanding input variables.

In recent years, machine learning and artificial intelligence have been increasingly integrated into the design and operation of smart buildings, with a growing focus on both energy sustainability and occupancy prediction. A representative example is presented in the study by^25^, which introduces an active learning-based framework for optimizing the energy performance of green buildings. The authors emphasize that although green buildings are designed to reduce environmental impact, occupant-related factors often cause them to consume up to 2.5 times more energy than planned. To address this issue, the study integrates advanced ML regressors such as Random Forest, Decision Tree, Gradient Boosting, XGBoost, CatBoost, LightGBM, KNN, and Logistic Regression. Through Z-Score normalization and active learning, the model achieved very high prediction accuracies of 0.9975 for cooling and 0.9883 for heating, showing that predictive modeling can drastically enhance energy efficiency. While the primary focus is on energy optimization, the results demonstrate the critical role of occupant presence and behavior in influencing building energy performance, providing indirect but highly relevant insights for occupancy detection research.

A more targeted contribution is offered by^26^, who present a comprehensive review of occupancy prediction in IoT-enabled smart buildings. This study highlights the rapid proliferation of Internet of Things (IoT) devices for collecting environmental data such as temperature, humidity, and $\hbox {CO}_2$ concentration, which can then be processed using ML techniques to infer occupant presence and behavior. The paper critically examines the strengths and weaknesses of various sensing technologies and predictive methods, ranging from traditional statistical models to advanced ML algorithms. Key challenges such as sensor placement, multimodal data fusion, scalability across different building types, and data privacy are thoroughly discussed. Importantly, the review emphasizes that smart occupancy prediction is central to reducing energy waste and ensuring efficient building management, making it directly aligned with the research on room-level occupancy detection using AI/ML/DL.

Focusing on deep learning approaches,^27^ developed an LSTM-based recurrent neural network to predict occupancy states in a multi-room setting. Recognizing that building energy consumption is closely tied not only to presence or absence but also to occupant behavioral patterns, the study trained RNNs using environmental inputs such as $\hbox {CO}_2$ levels, temperature, and humidity. The results demonstrated that recurrent neural networks are particularly well-suited for capturing temporal dependencies in occupancy data, enabling more accurate prediction of dynamic occupancy states. Unlike traditional methods that may require intrusive sensors or manual monitoring, this approach highlights the feasibility of non-intrusive, sensor-based AI techniques for smart buildings. This contribution is directly relevant, as it demonstrates how deep learning architectures can enhance occupancy detection performance in real-world environments.

In another application, though focused on healthcare,^28^ proposed a two-step Bayesian optimization framework for occupancy estimation in intensive care units (ICUs). The study employed XGBoost models optimized with Bayesian techniques to predict length of stay and occupancy levels in critical hospital settings. Although the context differs from residential or office buildings, the methodology is of particular interest: it illustrates how advanced optimization combined with ML can enhance prediction accuracy under conditions of high uncertainty and limited data. The scalability of this approach is also notable, as it demonstrates how occupancy-related ML models can be tailored to specialized environments with strict requirements. This makes the work indirectly relevant to room occupancy detection in smart buildings, especially in terms of methodological insights.

Directly addressing environmental sensing for occupancy,^29^ investigated the use of $\hbox {CO}_2$ concentration, differential pressure, and ventilation system data in occupancy estimation models. Employing artificial neural networks, the study showed that combining these environmental indicators provided a significant improvement in estimation accuracy compared to using $\hbox {CO}_2$ alone. This research not only highlights the strong correlation between indoor environmental quality and occupant presence but also emphasizes the importance of multi-sensor integration. Such findings are highly relevant to room occupancy detection in smart buildings, as they confirm that IoT-enabled sensing combined with ML/DL techniques can deliver robust predictions while also supporting broader applications such as indoor air quality management and infection risk reduction.

Finally,^30^ provide a systematic review of AI-driven big data analytics for building automation and management systems (BAMSs). The survey covers a wide range of AI applications in intelligent buildings, including load forecasting, energy anomaly detection, indoor environmental quality monitoring, and occupancy detection. By categorizing current approaches based on data sources, computational platforms, and application scenarios, the study reveals both the opportunities and limitations of integrating AI in smart building ecosystems. Importantly, it identifies occupancy detection as a key component of intelligent control systems, linking it directly to energy efficiency, user comfort, and system reliability. The paper also highlights the need for better data integration, scalability, and privacy-preserving approaches in future building automation solutions.

Table 1 summarizes recent occupancy detection and energy optimization research in smart buildings. While high accuracy is reported across different IoT-ML approaches, several limitations remain, as shown below.

**Table 1:** Summary of recent studies on occupancy detection and energy optimization with key methods, features, and limitations.

Ref.	Focus Area	Methods	Key Features	Limitations
^17^	IoT-based occupancy detection	RF, XGBoost, Dense NN	$\hbox {CO}_2$, temp., humidity, sound, light	Small dataset; limited generalization
^18^	PIR-based location/activity detection	SVM, PIR sensor array	Motion patterns, activity	Needs many sensors; high deployment cost; limited to motion
^19^	Multimodal occupancy prediction	Rule-based + interactive learning	Cameras + sensors	Privacy issues; limited scalability
^20^	Transfer learning for occupancy	Deep learning, normalization	Climate IoT sensors	Slightly lower accuracy for occupancy levels
^21^	Residential occupancy modeling	RF, BN, DT, SVM	IoT + survey data	Accuracy varies; privacy concerns
^22^	Room-level occupancy detection	XGBoost	Time, $\hbox {CO}_2$, dynamics	Limited transferability across buildings
^23^	Real-time detection (camera)	Faster R-CNN	Occupant count + activity	Lower accuracy for activities; privacy-sensitive
^24^	Occupancy + infection risk	ANN	$\hbox {CO}_2$, pressure, ventilation	Sensitive to door/window fluctuations
^25^	Energy sustainability in GB	Active learning + ML regressors	Energy + occupant-driven parameters	Focused on energy; occupancy only indirect
^26^	IoT-enabled occupancy prediction	Survey + comparative analysis	Sensor technologies, ML models	Conceptual; lacks implementation/experiments
^27^	Multi-room occupancy prediction	LSTM-RNN	Temporal patterns from env. data	Requires large training data; computationally expensive
^28^	ICU occupancy estimation	XGBoost + Bayesian optimization	Biomedical + monitoring data	Healthcare-specific; limited generalizability to buildings
^29^	$\hbox {CO}_2$-based occupancy estimation	ANN with ventilation data	$\hbox {CO}_2$, pressure, ventilation	Accuracy affected by airflow and sensor placement
^30^	AI in building automation	Systematic review + case studies	BAMS, occupancy, anomaly detection	Broad survey; no novel experimental model

In summary, while IoT-ML methods achieve high accuracy in predicting room occupancy, most studies face recurring limitations such as restricted datasets, lack of scalability across diverse building types, high computational costs, and privacy concerns when using cameras or sensitive environmental data. Addressing these limitations requires privacy-preserving frameworks, lightweight and generalizable models, and the development of large-scale open datasets to enable practical and reliable deployment of smart room occupancy detection systems.

## Materials and methods

**Fig. 1:**
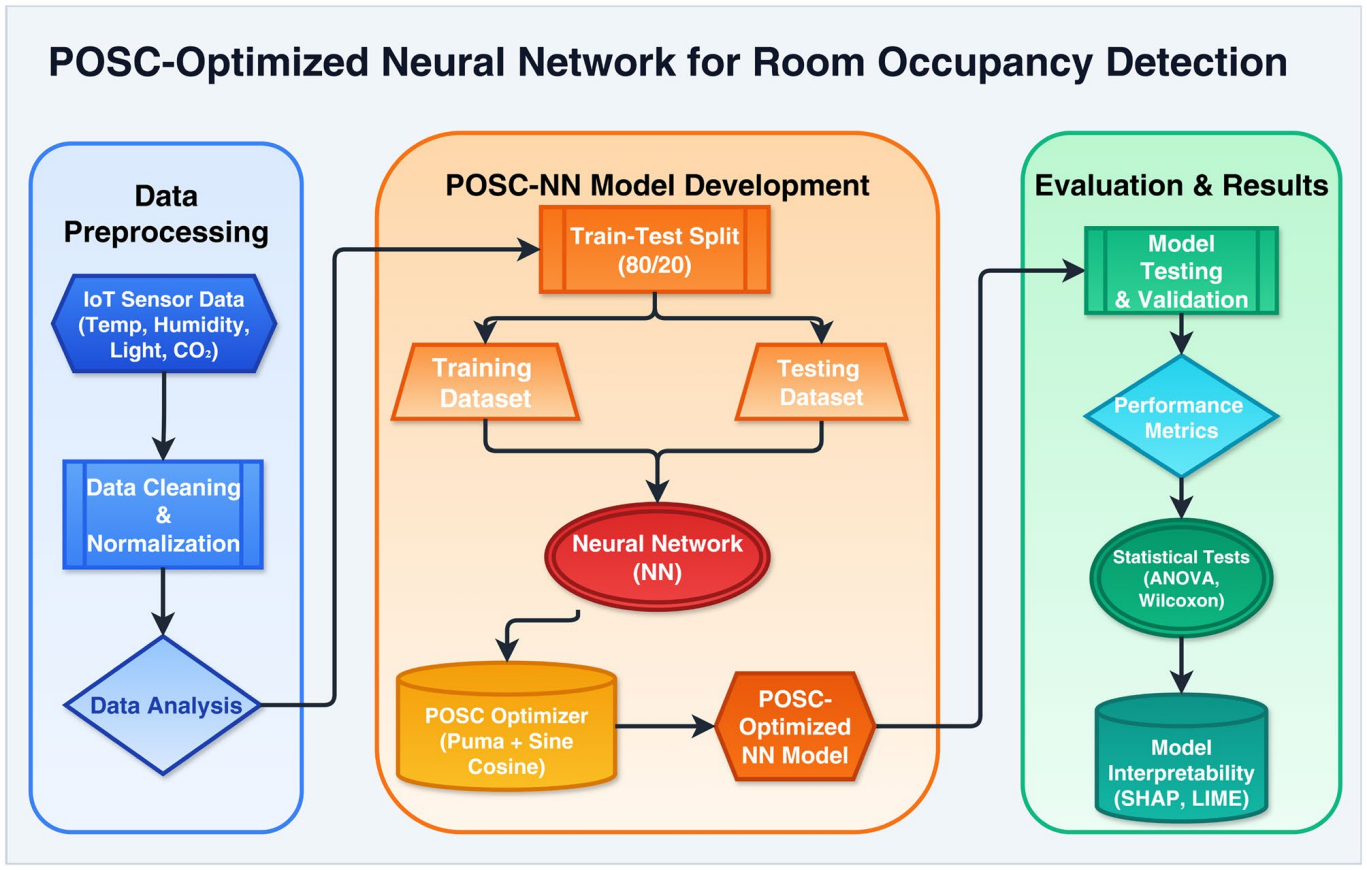
Proposed POSC-optimized Neural Network framework for room occupancy detection.

The proposed methodology for smart room occupancy detection follows a systematic three-stage framework that integrates data preprocessing, model development through hybrid optimization, and comprehensive evaluation, as illustrated in Fig. 1. The framework begins with data preprocessing, where environmental sensor data from IoT devices−including temperature, humidity, light intensity, and CO_2_ levels−are collected, cleaned, normalized, and analyzed for feature correlations before partitioning into training (80%) and testing (20%) subsets.

The second stage focuses on POSC-NN model development, where the Neural Network architecture is optimized using the hybrid Puma Optimizer and Sine Cosine Optimizer (POSC) algorithm that balances exploration and exploitation phases to minimize binary cross-entropy loss, accelerate convergence, and improve generalization while reducing overfitting risk. The final stage encompasses comprehensive evaluation through multiple performance metrics (accuracy, sensitivity, specificity, precision, NPV, and F1-score), statistical validation tests (ANOVA and Wilcoxon Signed Rank Tests) to compare POSC performance against GA and GWO algorithms, and interpretability analysis using SHAP and LIME techniques to ensure transparency and explainability for deployment in smart building automation and energy management systems.

### Dataset

The Room Occupancy Detection Data (IoT Sensor) dataset is built for binary classification, helping predict whether a room is occupied (1) or unoccupied (0) based on sensor readings. It includes four key features: Temperature ($^\circ$C), Humidity (%), Light (lux), and $\hbox {CO}_2$ Levels (ppm). The occupancy status was determined using timestamped images taken every minute, ensuring accurate labeling. This dataset is great for machine learning projects focused on smart buildings, energy efficiency, and automated occupancy detection. Since it uses environmental sensors rather than intrusive cameras, it’s useful for privacy-friendly monitoring solutions.

**Fig. 2:**
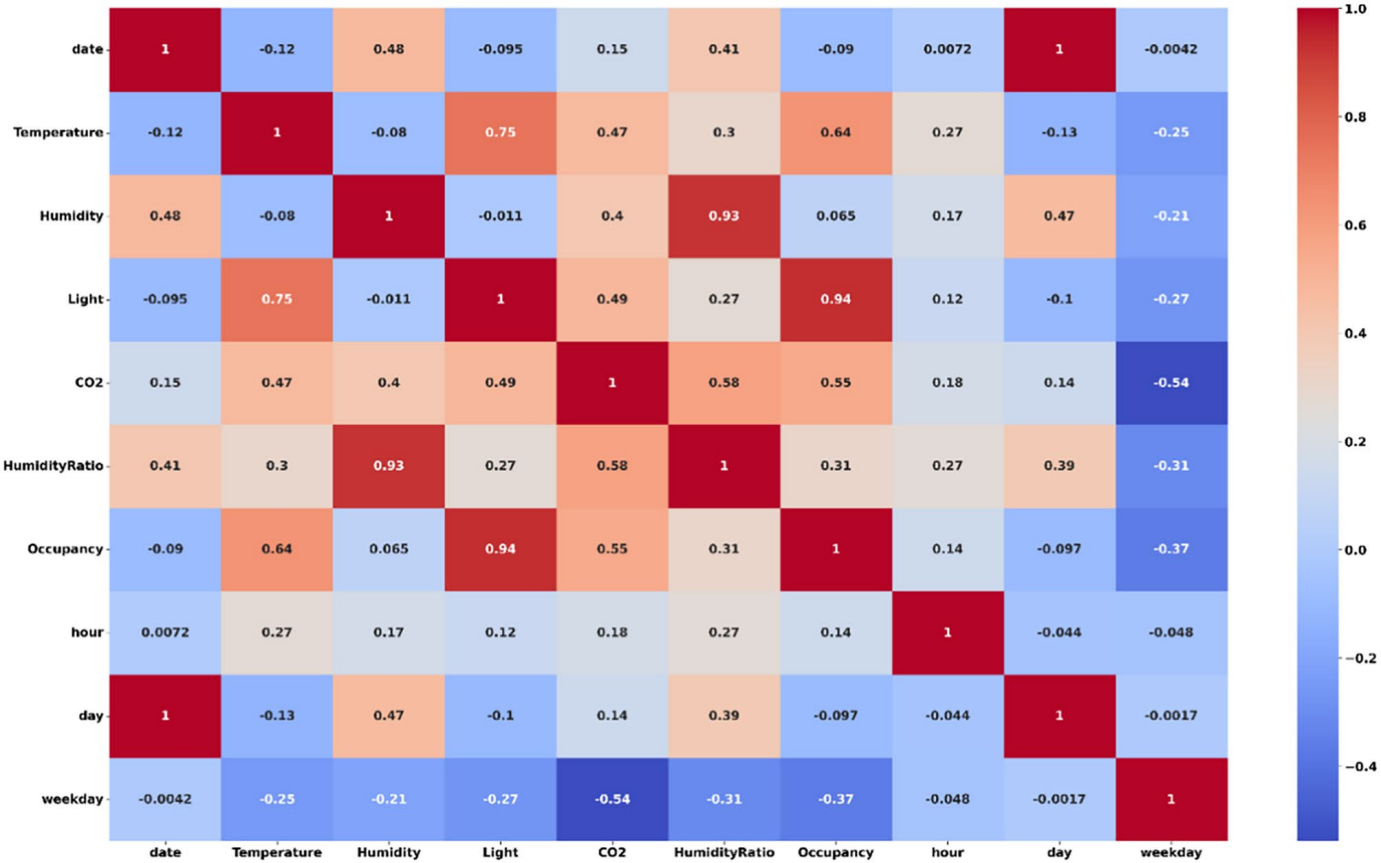
Correlation analysis of environmental features and room occupancy.

Figure 2 shows the correlation matrix of environmental parameters, time features, and occupancy status in a room environment. The heatmap highlights strong positive and negative correlations among features. Notably, light exhibits a strong positive correlation with occupancy, indicating its significant contribution to predicting whether the room is occupied. Temperature also has a noticeable positive correlation with occupancy, whereas humidity shows a weaker relationship. Humidity ratio correlates highly with humidity, as expected, due to its dependency on environmental moisture. The time-based features like hour, day, and weekday appear to have weaker correlations with occupancy, indicating minimal influence.

**Fig. 3:**
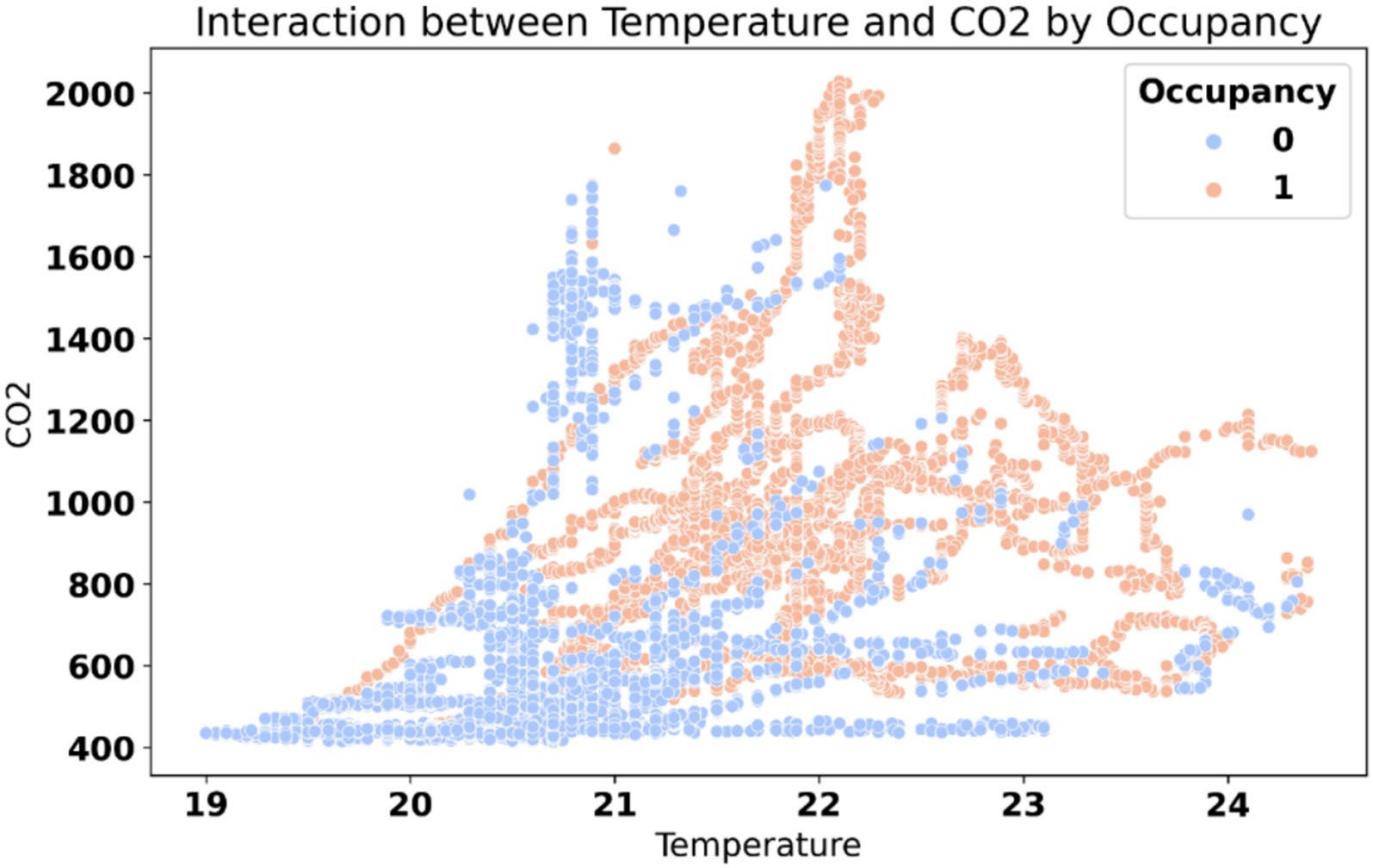
Scatter plot of temperature and $\hbox {CO}_2$ interaction by occupancy.

Figure 3 displays the interaction between temperature and $\hbox {CO}_2$ levels, differentiated by room occupancy status. Occupied instances (in orange) tend to cluster at higher $\hbox {CO}_2$ concentrations, indicating elevated human presence. These points also show a tendency toward higher temperatures, further supporting their correlation with occupancy. Conversely, unoccupied instances (in blue) are more scattered across lower $\hbox {CO}_2$ concentrations, reflecting reduced human activity and lesser influence on temperature levels. The overlap between clusters highlights instances where the temperature and $\hbox {CO}_2$ alone may not distinctly separate occupancy status. This interaction underlines the importance of combining multiple features for accurate occupancy detection.

The dataset reveals critical feature relationships for occupancy detection. Light exhibits the strongest correlation with occupancy, while temperature shows positive correlation due to body heat and device usage. Humidity and time-based features demonstrate weaker influences, suggesting schedule-independent occupancy patterns. Although $\hbox {CO}_2$ and temperature are strong indicators, combining multiple features (light, temperature, humidity, and $\hbox {CO}_2$) significantly improves prediction accuracy for intelligent building management applications.

### Neural network

Neural Networks are powerful machine learning models that excel at capturing complex patterns in data, making them highly effective for room occupancy detection. In this study, a feedforward neural network (FNN) is employed to classify room occupancy based on temperature, humidity, light, and $\hbox {CO}_2$ levels. The network consists of an input layer, multiple hidden layers, and an output layer, where each neuron processes data through weighted connections and activation functions.

The input layer takes in the four environmental sensor features:

$$\begin{aligned} X = [x_1, x_2, x_3, x_4] = [\text {Temperature}, \text {Humidity}, \text {Light}, \text {CO}_2] \end{aligned}$$

Each neuron in the hidden layers processes the input using weighted connections and a bias term:

$$\begin{aligned} z_j = \sum _{i=1}^{n} w_{ij}x_i + b_j \end{aligned}$$

where $z_j$ is the weighted sum of inputs for neuron $j$, $w_{ij}$ represents the weight between input $x_i$ and neuron $j$, $b_j$ is the bias term, and $n$ is the number of input features.

The activation function ReLU (Rectified Linear Unit) introduces non-linearity:

$$\begin{aligned} a_j = \max (0, z_j) \end{aligned}$$

For the output layer, a sigmoid activation function produces the probability of occupancy:

$$\begin{aligned} \hat{y} = \frac{1}{1 + e^{-z_k}} \end{aligned}$$

where $\hat{y}$ is the predicted probability of the room being occupied, and $z_k$ is the weighted sum of activations from the last hidden layer.

The final classification decision is based on:

$$\begin{aligned} y = {\left\{ \begin{array}{ll} 1, & \text {if } \hat{y} \ge 0.5\\ 0, & \text {otherwise} \end{array}\right. } \end{aligned}$$

### Training and optimization using POSC

The NN is trained using supervised learning with labeled occupancy data. Instead of conventional gradient-based optimizers, this study employs the hybrid Puma Optimizer-Sine Cosine Optimizer (POSC) to enhance convergence speed, generalization, and classification accuracy.

The Puma Optimizer (PO)^31^ simulates the adaptive hunting strategy of pumas by alternating between exploration (searching new areas) and exploitation (refining solutions). The Sine Cosine Optimization (SC)^32^ complements this with sinusoidal motion to improve convergence and maintain diversity.

The fitness function is the binary cross-entropy loss, defined as:

$$\begin{aligned} L = -\frac{1}{m} \sum _{i=1}^{m} [y_i \log (\hat{y_i}) + (1 - y_i)\log (1 - \hat{y_i})] \end{aligned}$$

where $m$ is the number of samples, $y_i$ the true label, and $\hat{y_i}$ the predicted probability.

PO alternates exploration and exploitation phases, quantified as:

$$\begin{aligned} Score_{Explore}= & (PF1 \cdot f1_{Explor}) + (PF2 \cdot f2_{Explor})\\ Score_{Exploit}= & (PF1 \cdot f1_{Exploit}) + (PF2 \cdot f2_{Exploit}) \end{aligned}$$

where PF1, PF2 are weighting factors, and $f1, f2$ are exploration/exploitation cost functions.

In the Sine Cosine phase, weights are updated as:

$$\begin{aligned} P(t+1) = {\left\{ \begin{array}{ll} P(t) + r_5 \cdot \sin (r_6) \cdot |r_7 S^*(t) - S(t)|, & \text {if } r_4 < 0.5\\ P(t) + r_5 \cdot \cos (r_6) \cdot |r_7 S^*(t) - S(t)|, & \text {if } r_4 \ge 0.5 \end{array}\right. } \end{aligned}$$

where $P(t)$ is the current weight vector, $S^*(t)$ is the best solution, and $r_4, r_5, r_6, r_7$ are random numbers introducing diversity.

This adaptive mechanism balances exploration and exploitation for faster convergence and improved accuracy, as illustrated in Algorithm 1.

**Algorithm 1:**
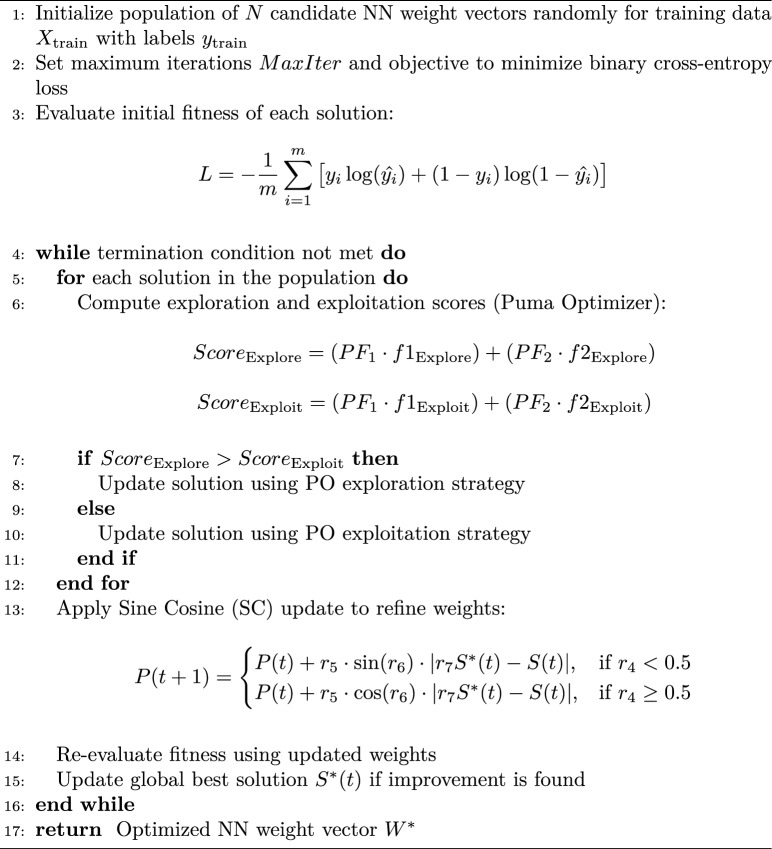
POSC-optimized neural network training.

### Evaluation metrics

The trained model is evaluated on unseen data using standard classification metrics. Table 2 summarizes the mathematical formulations of accuracy, sensitivity, specificity, precision, negative predictive value (NPV), and the F1-score^33^.

**Table 2:** Classification performance indicators.

Metric	Formula
Accuracy	$\frac{TP + TN}{TP + TN + FP + FN}$
Sensitivity (TPR)	$\frac{TP}{TP + FN}$
Specificity (TNR)	$\frac{TN}{TN + FP}$
Precision (PPV)	$\frac{TP}{TP + FP}$
Negative Predictive Value (NPV)	$\frac{TN}{TN + FN}$
F1-score	$2 \times \frac{\text {Precision} \times \text {Recall}}{\text {Precision} + \text {Recall}}$

Integrating PO and SC optimization significantly enhances the NN’s generalization across different occupancy scenarios. Compared to other optimization methods, the POSC-optimized NN achieves superior classification accuracy, faster training, and robustness against overfitting, validating its potential for smart building energy management and intelligent occupancy detection.

## Experimental results

This section presents the experimental evaluation of the proposed POSC-optimized Neural Network for room occupancy detection. The baseline Neural Network model demonstrated promising performance prior to optimization, achieving strong sensitivity and specificity rates that indicate effective discrimination between occupied and unoccupied states. These initial results established a solid foundation for further enhancement through metaheuristic optimization. To ensure statistical robustness and reliability, all experiments were repeated ten times with different random weight initializations. This repetition strategy mitigates the impact of stochastic variation inherent in neural network training and provides confidence intervals for performance metrics. Comparative analysis employing one-way ANOVA and Wilcoxon Signed Rank Tests confirmed that the POSC optimization significantly improved model performance across all evaluation criteria. These findings demonstrate that hybrid metaheuristic algorithms can effectively enhance deep learning models for intelligent building automation applications.

### Neural network architecture

Table 3 details the architecture of the feedforward neural network employed in this study. The network accepts four environmental sensor inputs (temperature, humidity, light intensity, and $\hbox {CO}_2$ concentration) through the input layer. Two hidden layers with 64 and 32 neurons respectively, both utilizing ReLU activation functions, enable the network to learn complex non-linear relationships between environmental features and occupancy status. The output layer employs a single neuron with sigmoid activation to produce binary occupancy predictions. This architecture encompasses 4,225 trainable parameters, representing a balance between model capacity and computational efficiency suitable for real-time deployment in IoT-enabled smart buildings.

**Table 3:** Neural network architecture specifications.

Layer	Specifications
Input Layer	4 neurons (Temperature, Humidity, Light,$\hbox {CO}_2$ levels)
Hidden Layer 1	64 neurons with ReLU activation function
Hidden Layer 2	32 neurons with ReLU activation function
Output Layer	1 neuron with Sigmoid activation function
Total Parameters	4,225 trainable parameters

Table 4 provides a detailed breakdown of parameter distribution across network layers. The largest concentration of parameters resides in the connection between Hidden Layer 1 and Hidden Layer 2, comprising 2,048 weight parameters and 32 bias terms for a total of 2,080 parameters. This layer accounts for nearly half of the network’s total capacity (49.2%), reflecting its central role in feature transformation. Weight parameters constitute 55.3% of the total (2,336 out of 4,225), while bias parameters represent 2.3% (97 parameters). This distribution analysis provides transparency regarding model complexity and facilitates understanding of computational requirements for the POSC optimization process.

**Table 4:** Neural network parameter distribution summary.

Layer Connection	Weight Parameters	Bias Parameters	Total
Input $\rightarrow$ Hidden 1	4 $\times$ 64 = 256	64	320
Hidden 1 $\rightarrow$ Hidden 2	64 $\times$ 32 = 2,048	32	2,080
Hidden 2 $\rightarrow$ Output	32 $\times$ 1 = 32	1	33
Total	2,336	97	4,225

### Machine learning models results

Table 5 describes the performance of the best model, and it shows that by using NN, the sensitivity (TPR) is 0.95137 while the specificity (TNR) is 0.96226. Such findings clearly illustrate that the proposed NN model provides very high levels of accuracy, where the high sensitivity means that the model can accurately identify most of the actual positive cases. The high specificity as such proves the model’s ability to isolate negative cases and thereby improves the reliability and the overall classification performance of the model.

**Table 5:** Evaluation metrics of machine learning models.

Model	Accuracy	Sensitivity	Specificity	PPV	NPV	F1-score
Neural Network (NN)	0.95712	0.95137	0.96226	0.95745	0.95685	0.95440
Support Vector Machine (SVM)	0.92795	0.91884	0.93620	0.92879	0.92720	0.92379
K-Nearest Neighbors (K-NN)	0.91679	0.90698	0.92567	0.91693	0.91667	0.91193
Naïve Bayes (NB)	0.90604	0.89623	0.91489	0.90476	0.90717	0.90047

**Fig. 4:**
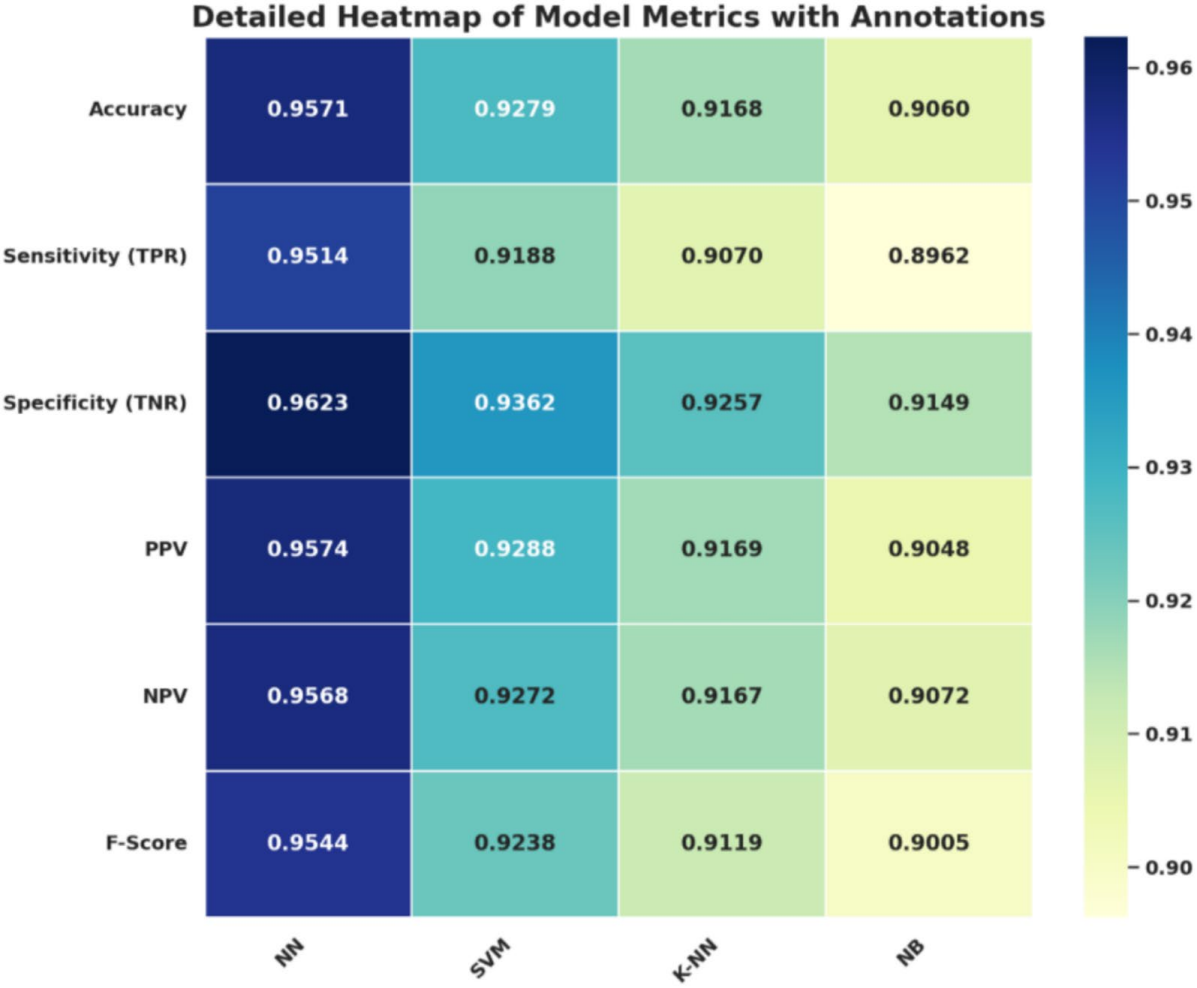
Heatmap of model metrics for comparative performance analysis.

To provide a better understanding of the various evaluation metrics, a more detailed heatmap with additional metrics is presented in Fig. 4, in which the NN has emerged as the most promising model. NN also generates better performance in every efficiency indication, including accuracy, sensitivity, specificity, PPV, NPV, and F-score. The conformity of the deep blue color, except for NN, across all its measured metrics gives it a clear advantage over other models as it entails high instance classification with both a high level of precision and recall. The fact that NN has demonstrated consistent results across the metrics proves that it can generalize whether the output values are positive or negative. The heatmap also enhances the comparison between higher results by the NN model to lower-performing models, displaying the discriminative ability of the model on classification tasks.

**Fig. 5:**
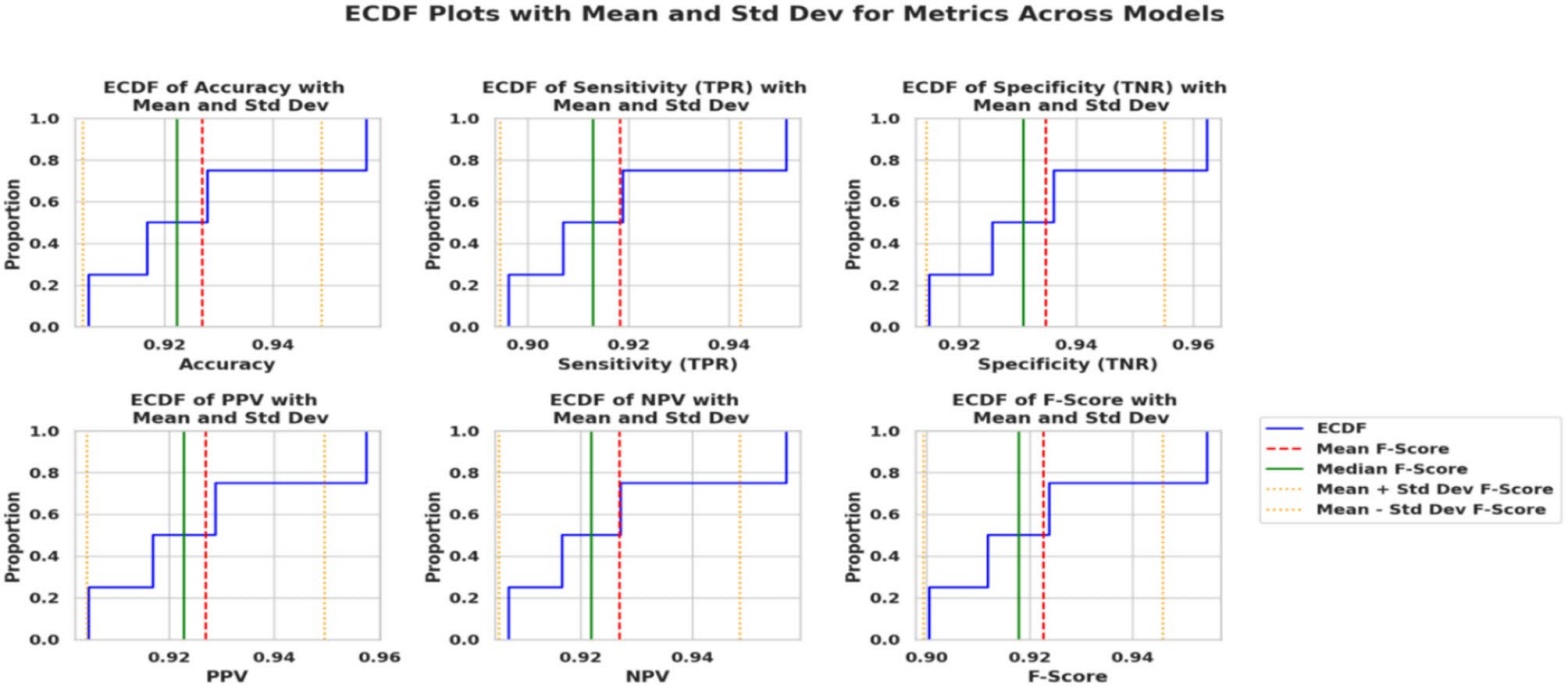
ECDF analysis of metrics for model performance evaluation.

Figure 5 presents the Empirical cumulative distribution function (ECDF) plots of all seven chosen model metrics with accuracy, sensitivity, specificity, positive predictive value, negative predictive value, as well as F1 score out and across the models. Raw cohort and time series data are depicted by red curves, and the blue ECDF curves show the metric values of the distribution of the dynamics, with referent mean, median, and standard deviations marked. The curves of all the models rise steeply near the upper thresholds, indicating that the NN, which is the best model, is bound closer to the higher metric values. As shown in this figure, it shows how the proposed NN model is superior and dominant in providing better classification performance consistently. The alignment of the obtained curve with the statistical thresholds strengthens the conclusion about the balanced and equally optimal performance of the NN in each of the evaluated criteria.

### Optimization results

Table 6 depicts the performance results of the POSC + NN model, where accuracy equals 0.98059. These results provide a clear picture of the performance of the POSC + NN model for this new set of samples, which clearly demonstrates a high level of accuracy, as evidenced by the fact that most instances have been classified accurately by the POSC + NN model. The high F-score reinforces the fairly accurate estimate of both precision and recall, which in this case confirms non-optimality and precise fit for the designed model.

**Table 6:** Performance evaluation of optimized NN models.

Models	Accuracy	Sensitivity	Specificity	PPV	NPV	F1-Score
POSC + NN	0.98059	0.97755	0.98329	0.9811	0.98014	0.97933
PO + NN	0.975774	0.972850679	0.980066445	0.986239	0.960912	0.979499
SC+NN	0.970549	0.964125561	0.980066445	0.986239	0.948553	0.975057
GA + NN	0.96663	0.96257	0.97028	0.9667	0.96657	0.96463
GWO + NN	0.95593	0.95106	0.96028	0.95531	0.95648	0.95318

**Fig. 6:**
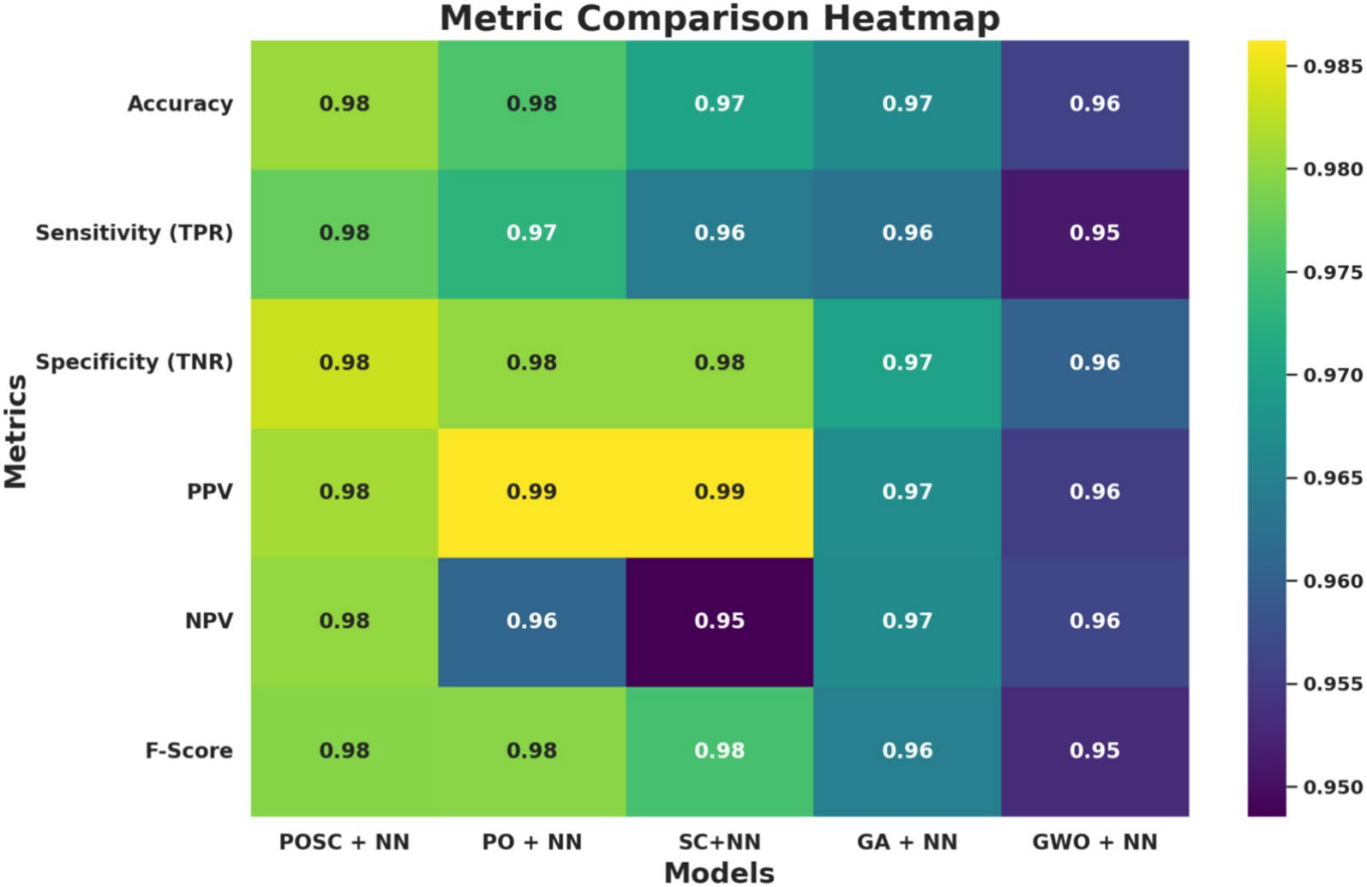
Heatmap of metrics for optimized NN models.

Figure 6 gives a detailed heatmap showing various evaluation metrics for the optimized NN model and shows that POSC + NN outperforms all given models. In all the proposed performance indicators, namely accuracy, sensitivity, specificity, PPV, NPV, and F-score, the POSC + NN model yields the highest figure throughout the analysis. The darker blue color applied to POSC + NN indicates its performance advantage in greater interpretation of true positive and true negative data, with the trade-off of precision and recall. Nonetheless, the lighter densities of GA + NN and GWO + NN slightly lag these performances across this set of metrics.

**Fig. 7:**
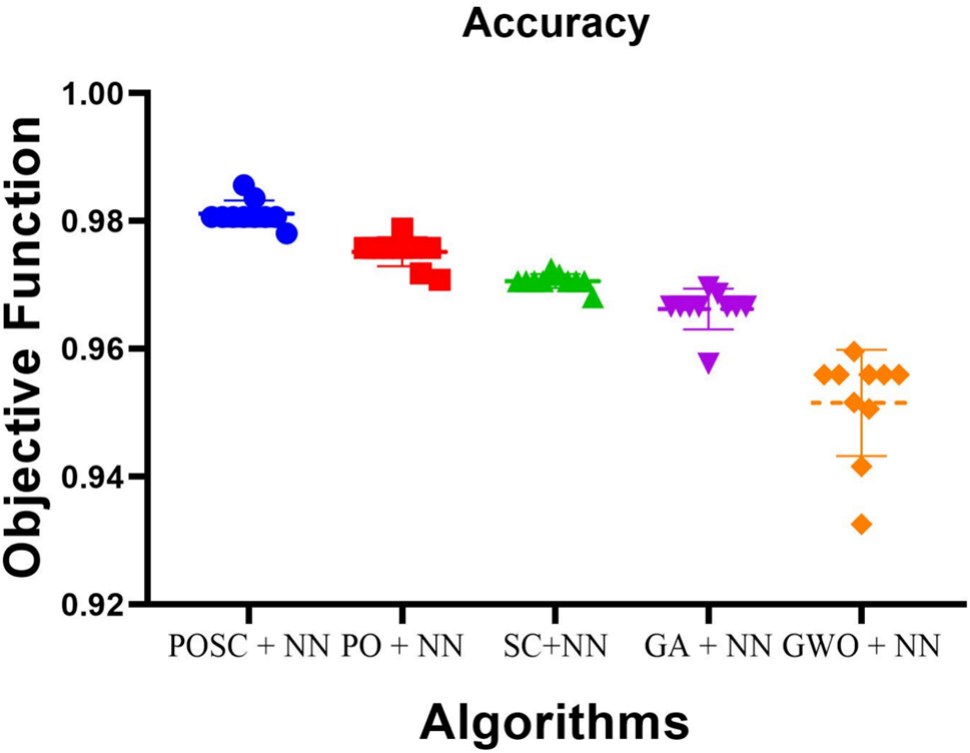
Accuracy comparison of optimization algorithms with Neural networks.

Figure 7 illustrates the comparative accuracy of different optimization algorithms paired with neural networks (NN). The algorithms, combining optimization techniques with NN, are displayed along the horizontal axis, while accuracy is measured on the vertical axis. Each point represents an accuracy value achieved across multiple runs. Among the models, POSC+NN achieves the highest accuracy, with values reaching 0.98 and a median around 0.96. This tight clustering of results demonstrates both the stability and reliability of the POSC-based optimization process, highlighting its superior performance relative to other methods.

**Fig. 8:**
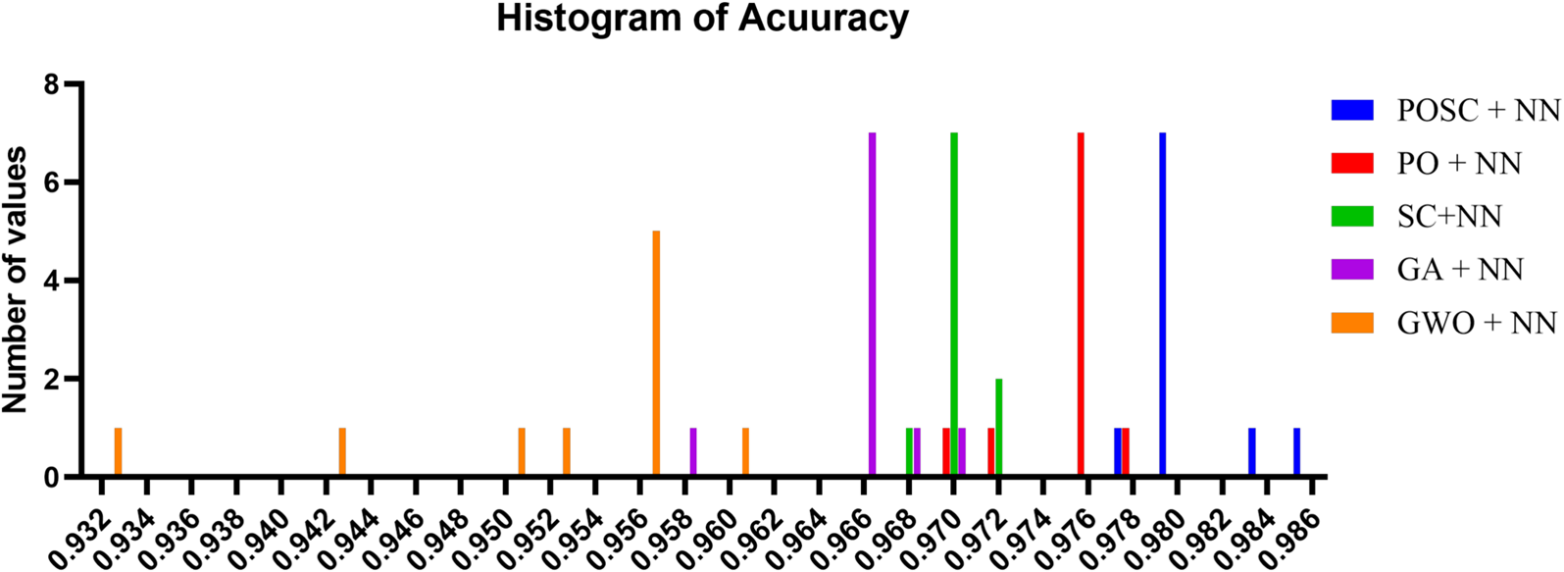
Accuracy value distribution of optimization-neural network models.

Figure 8 presents the distribution of accuracy values obtained from different optimization-NN hybrid models. The histograms capture the spread and concentration of performance outcomes across repeated runs, highlighting the stability and robustness of each algorithm. POSC+NN demonstrates a narrower and more concentrated distribution around higher accuracy levels, indicating reliable convergence toward optimal solutions. In contrast, other algorithms show wider spreads, suggesting greater variability in their optimization outcomes. This analysis underscores the consistency and effectiveness of the POSC+NN approach compared to alternative models.

Figure 16 illustrates a comparative analysis of various algorithms, focusing on their detection capabilities. The key metrics used for evaluation are detection rate, false positive rate, and false negative rate, assessing enhancements over a base model achieved through different optimization techniques. Among the algorithms tested, ’POSC + NN’ demonstrates superior performance across the board. Specifically, ’POSC + NN’ achieves a noteworthy result in Detection Rate, additionally ’POSC + NN’ also shows a favorable False positive rate, further reinforcing its effectiveness.

**Fig. 9:**
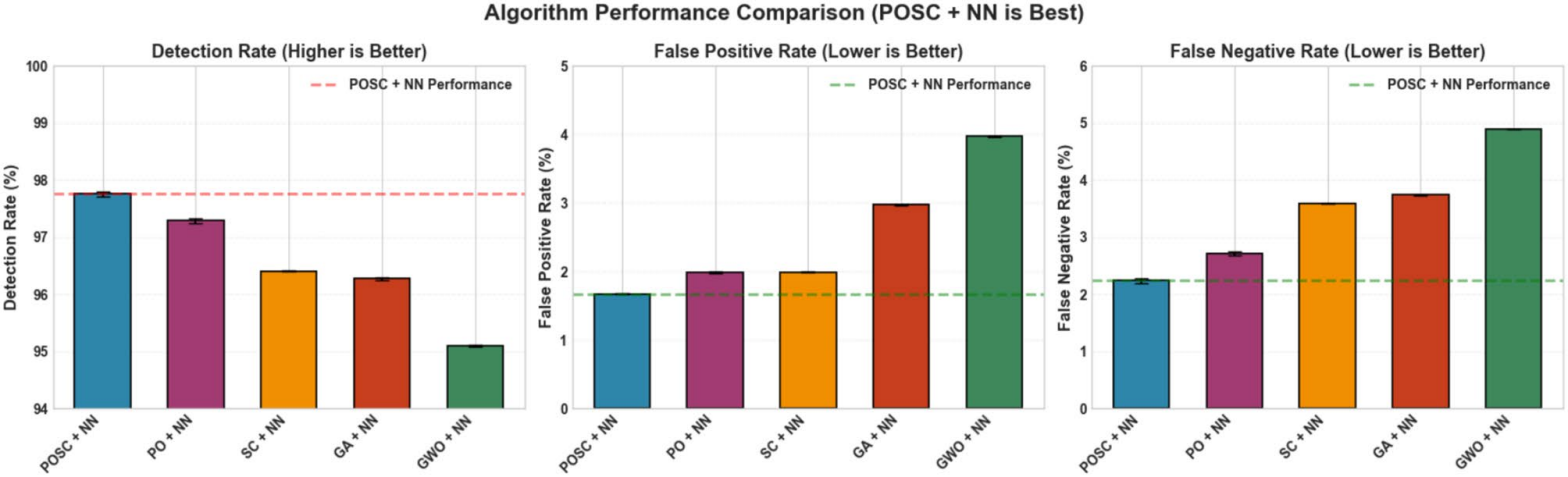
Performance comparison of optimization algorithms for a POSC + NN model.

### Statistical analysis

Table 7 presents the results of the ANOVA test. The analysis partitions the total sum of squares (SS) into treatment (between-group) and residual (within-group) components, allowing for the evaluation of variance explained by treatment effects. The findings reveal a significant treatment effect, with an F-statistic of 69.67, $F(4, 45)$, indicating substantial differences among the treatment groups. The sum of squares for the treatment effect is 0.004997, with a corresponding mean square (MS) of 0.001249. The residual sum of squares is 0.000807 with 45 degrees of freedom (DF), while the total DF equals 49. The associated p-value is less than 0.0001, confirming that the observed differences across treatments are highly statistically significant.

**Table 7:** ANOVA results for performance comparison of optimized-neural network algorithms.

ANOVA table	SS	DF	MS	F (DFn, DFd)	P value
Treatment (between columns)	0.005	4	0.0012	F (4, 45) = 69.67	P<0.0001
Residual (within columns)	0.0008	45	0.0		
Total	0.0058	49			

Table 8 presents a comparative analysis of different optimization models using parameters related to statistical significance. The results are reported from the Wilcoxon Signed Rank Test, including theoretical and actual medians, number of values, sums of signed, positive, and negative ranks, as well as p-values. The table also indicates whether the p-value is exact or estimated, provides a p-value summary, and assesses significance at the $\alpha = 0.05$ level. A discrepancy metric is included to quantify differences between models, offering a comprehensive evaluation. Among the models tested, POSC+NN demonstrates the best performance, with a discrepancy value of 0.9806 and a p-value of 0.002. This result is statistically significant at $\alpha = 0.05$.

**Table 8:** Wilcoxon signed-rank test results for median comparison - Sheet3.

Metric	POSC + NN	PO + NN	SC + NN	GA + NN	GWO + NN
Theoretical median	0	0	0	0	0
Actual median	0.9806	0.9758	0.9705	0.9666	0.9559
Number of values	10	10	10	10	10
**Wilcoxon Signed-Rank Test**					
Sum of signed ranks (W)	55	55	55	55	55
Sum of positive ranks	55	55	55	55	55
Sum of negative ranks	0	0	0	0	0
P value (two-tailed)	0.002	0.002	0.002	0.002	0.002
Exact or estimate?	Exact	Exact	Exact	Exact	Exact
P value summary	**	**	**	**	**
Significant (alpha = 0.05)?	Yes	Yes	Yes	Yes	Yes
**Discrepancy**					
Discrepancy value	0.9806	0.9758	0.9705	0.9666	0.9559

**Fig. 10:**
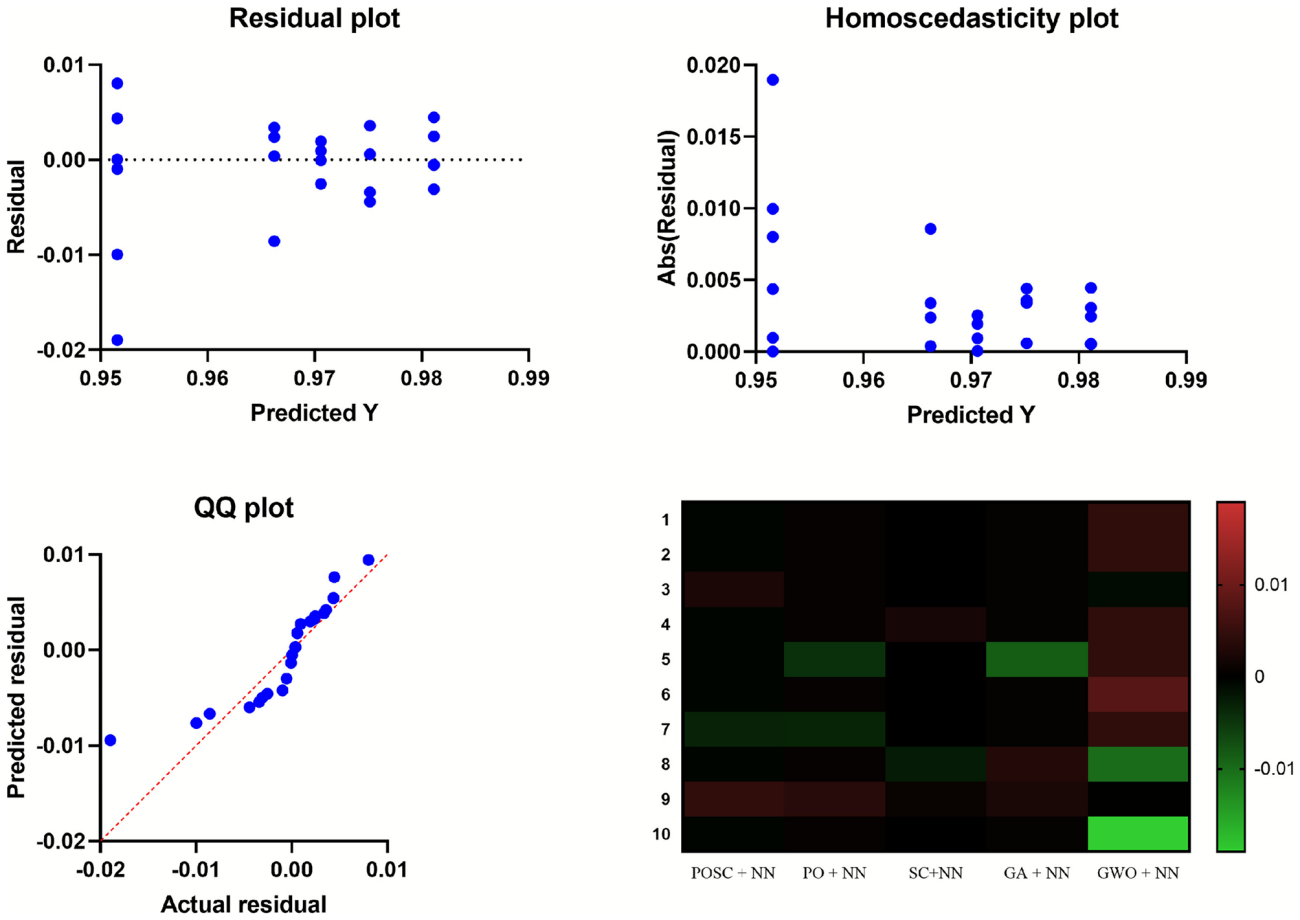
Comprehensive performance evaluation of the optimized-neural network algorithms.

Figure 10 demonstrates the statistical outcomes for POSC + NN, PO + NN, SC + NN, GA + NN, and GWO + NN. The figure provides distribution charts, performance scatter plots, and a heatmap for pairwise comparison. All testing conditions demonstrate that POSC + NN consistently delivers stable and superior performance values. Visual patterns in the results demonstrate that POSC + NN maintains minimal variation while achieving the most accurate expected performance values across all assessment tests. The data obtained from the heatmap demonstrate that POSC + NN delivers the highest consistency with accuracy, while surpassing other models in every performance aspect.

### Convergence analysis

**Fig. 11:**
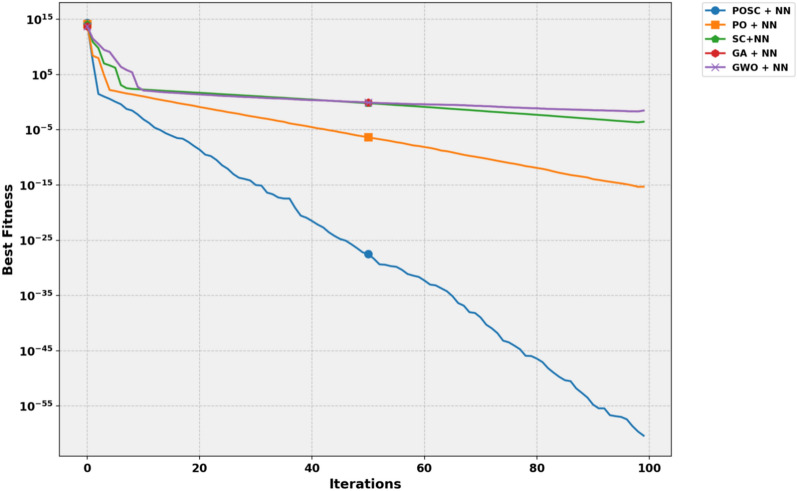
Convergence analysis of metaheuristic-optimized neural network models.

Figure 11 shows the convergence behavior of POSC + NN, PO + NN, SC + NN, GA + NN, and GWO + NN across 100 iterations. The Y-axis (logarithmic scale) indicates the best fitness value, and the X-axis represents the number of iterations. POSC + NN demonstrates the most rapid and significant decline in fitness values, indicating superior convergence performance. Compared to other methods, it consistently achieves lower fitness levels, highlighting its effectiveness in optimizing NN training by maintaining a strong balance between exploration and exploitation.

**Fig. 12:**
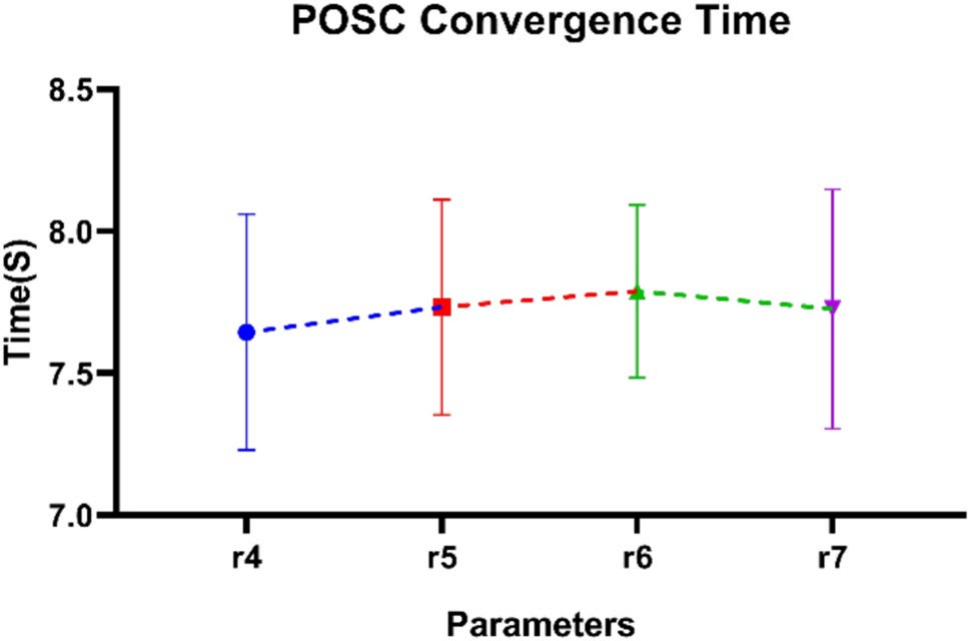
POSC Algorithm Convergence Time Analysis Across Parameter Configurations.

Figure 12 indicates the convergence time of the POSC algorithm in various parameter settings. The findings consistently show convergence behavior, with the timing being stable compared to all the sets of parameters involved in the test. The performance of each parameter set is nearly identical, with a slight difference in convergence time. It implies that the algorithm’s behavior is expected to be reliable, as the error bars indicate that the convergence process is acceptable in terms of disparity. The cumulative trend indicates that POSC has remained effective despite the selection of parameters. The consistency observed in various setups demonstrates that the proposed optimization strategy is robust.

**Fig. 13:**
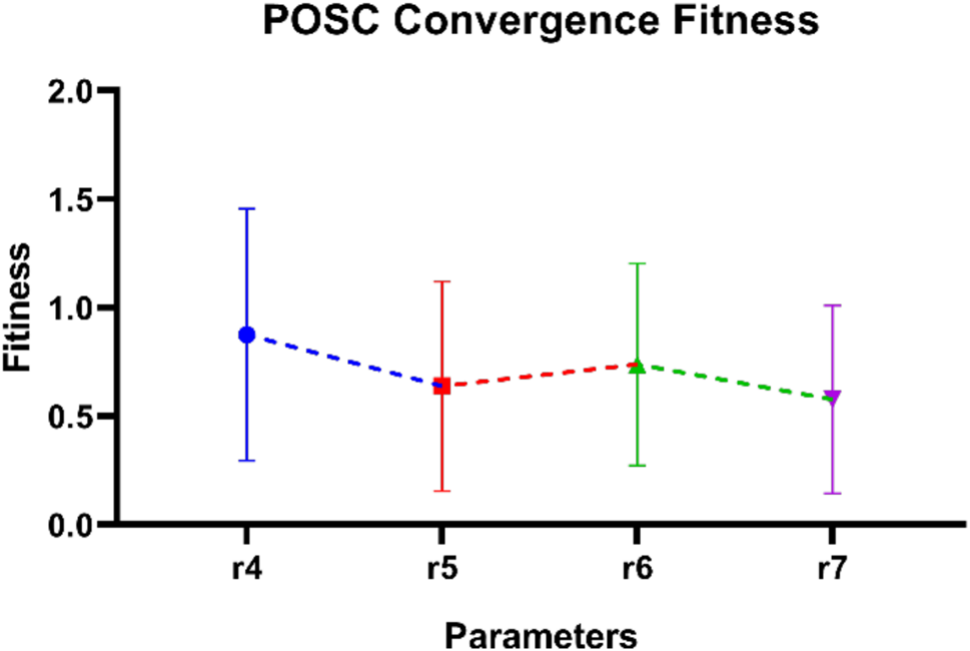
POSC Algorithm Convergence Fitness Performance Across Parameter Settings.

Figure 13 illustrates the convergence fitness achieved by the POSC algorithm at various parameter settings. The results are plotted to illustrate optimization performance against final fitness values after convergence. The individual fit differs in each parameter configuration, but both demonstrate good minimization ability. Error bars indicate the range of variation in final solutions observed across repeated simulations for each parameter set. Fitness values indicate the optimal activity on all tested setups. The reliability of POSC in attaining optimal or near-optimal solutions can be demonstrated by the consistently high levels of fitness achieved whenever it is run.

**Fig. 14:**
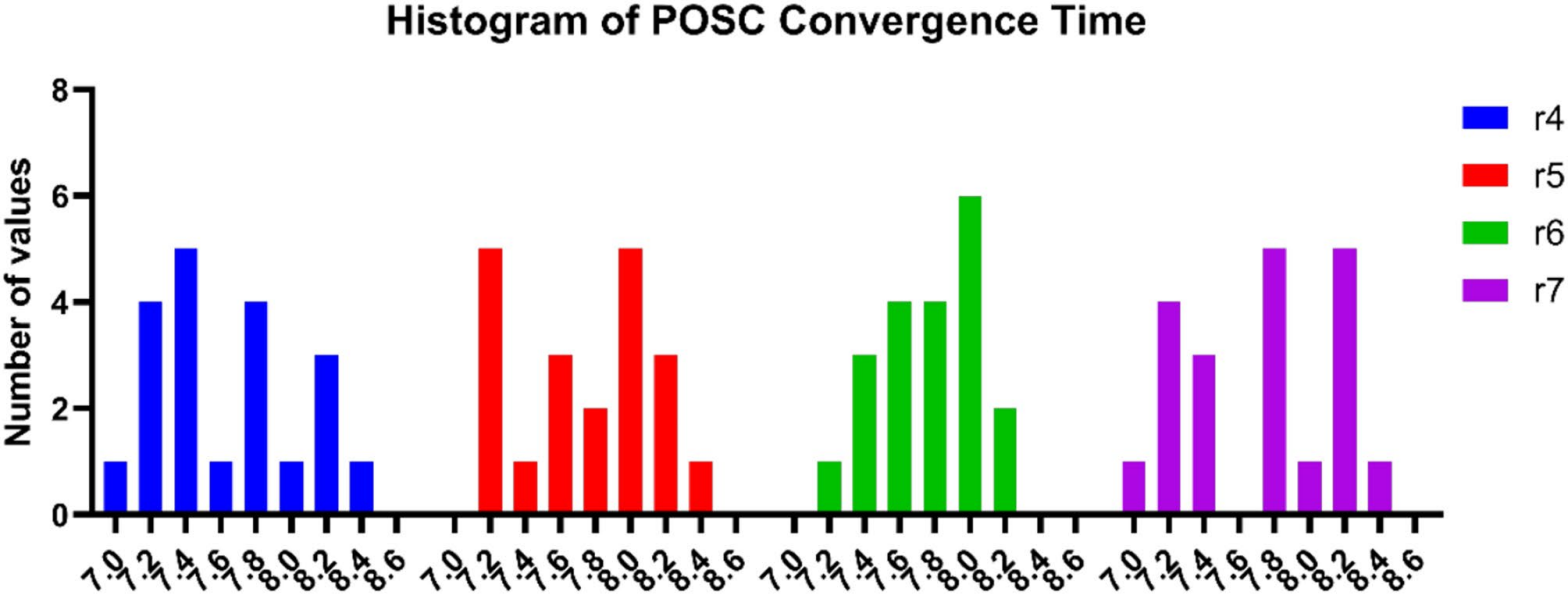
Distribution analysis of POSC convergence time across parameter configurations.

In Fig. 14, the histograms of convergence times of the POSC algorithm in different parameter sets are analyzed. Every parameter combination has different distribution documents regarding the timing frequency of convergence. The histograms indicate the focus of convergence times within a given range for each parameter setup. Patterns and distorts in distributions exhibit some consistency in convergence behavior on different parameter settings. A certain setup exhibits a more focused distribution, whereas others have wider distributions in terms of convergence timing. The analysis of histograms will introduce the data regarding the reliability and predictability of the convergence performance per parametrical set.

**Fig. 15:**
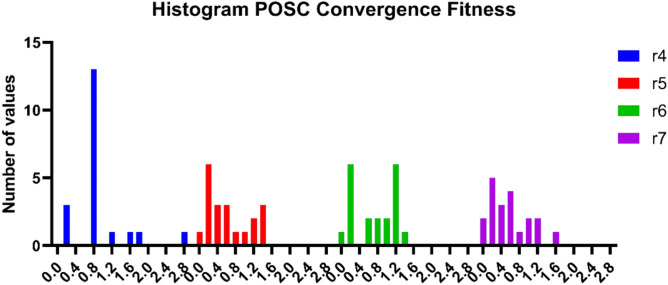
Fitness value distribution analysis for POSC algorithm across parameter settings.

Figure 15 demonstrates the frequency of fitness values obtained when using the POSC algorithm in various parameter settings. The histograms display the distribution of fitness values across multiple runs of the optimization algorithm within a single parameter set. All the parameter settings have different patterns of distribution for the value of fitness and varying concentrations of regions near the optimum. As the distribution analysis shows, with multiple runs, it is possible to note the uniformity of optimization performance, and the algorithms manage to reach close fitness levels. On the one hand, there are parameter sets that are more dense around certain fitness intervals, and on the other hand, there are those that are more widespread. Histogram trends are very helpful when gauging the reliability and stability of POSC optimization performance.

Table 9 presents the performance of the POSC algorithm in terms of convergence time across four parameter setups, characterized by varying parameter values. The results indicate the timing characteristics of the algorithm when various parameters were used, as well as the relationship between parameter variation and the convergence rate. The parameter configuration used at each parameter setting has a set of various convergence times depending on the parameter settings, implying susceptibility to parameter tuning. The convergence times are all within acceptable ranges across all sets of parameters, indicating that the algorithm is well-behaved and should not underperform for specific parameter choices. Others exhibit more regular timing with certain parameter set configurations, while others feature more diverse timing dynamics as parameter values vary. The exhaustive parameter scan provides clues regarding the temporal efficiency of the algorithm, as well as assistance in finding an optimal parameter regime for practical utilization.

**Table 9:** Convergence time results for different values of POSC’s parameters.

r4		r5		r6		r7	
Values	Time	Values	Time	Values	Time	Values	Time
0.1	7.765	0.05	7.214	0.1	7.895	0.05	7.162
0.2	8.063	0.1	8.107	0.2	7.941	0.1	7.368
0.3	8.258	0.15	7.551	0.3	8.100	0.15	7.453
0.4	7.838	0.2	8.036	0.4	8.078	0.2	7.873
0.5	7.441	0.25	8.050	0.5	7.440	0.25	7.758
0.6	7.871	0.3	7.235	0.6	7.623	0.3	8.112
0.7	7.088	0.35	7.936	0.7	7.213	0.35	8.373
0.8	7.202	0.4	7.286	0.8	7.740	0.4	7.285
0.9	7.460	0.45	7.442	0.9	7.630	0.45	8.295
1.0	8.201	0.5	8.323	1.0	8.024	0.5	7.079
1.1	8.128	0.55	7.110	1.1	8.054	0.55	7.758
1.2	7.164	0.6	8.148	1.2	7.700	0.6	8.195
1.3	7.879	0.65	8.115	1.3	7.637	0.65	7.999
1.4	7.322	0.7	7.849	1.4	7.411	0.7	7.724
1.5	7.401	0.75	7.687	1.5	7.828	0.75	7.228
1.6	8.401	0.8	7.814	1.6	8.294	0.8	7.458
1.7	7.659	0.85	7.175	1.7	8.255	0.85	8.242
1.8	7.163	0.9	7.621	1.8	7.415	0.9	8.102
1.9	7.278	0.95	7.953	1.9	7.509	0.95	7.881
2.0	7.316	1.0	8.003	2.0	7.995	1.0	7.190

Table 10 illustrates the fitness optimization success of the POSC algorithm under the same set of four parameter settings used to analyze convergence. Values of fitness show the optimizability of the algorithm with variations in the parameters, indicating the sensitivity of changing the parameters to the quality of solutions. The distinct parameter settings yield different fitness results at various parameter values, suggesting that optimization performance is compromised by tuning parameters. The performance on fitness indicates that POSC can perform well as an optimizer, even in a variety of parameter settings; however, certain parameter values consistently outperform others. Some parameter sets yield good minimization results with low fitness values, while others exhibit optimization results with greater variance. A thorough fitness analysis provides vital information about parameter selection and performance prediction in parameter optimization, particularly in real-life settings.

**Table 10:** Minimization results for different values of POSC’s parameters,

r4		r5		r6		r7	
Values	Fitness	Values	Fitness	Values	Fitness	Values	Fitness
0.1	0.7084	0.05	0.22144704	0.1	0.729656	0.05	1.062499
0.2	0.174	0.1	0.46911106	0.2	1.267736	0.1	0.861331
0.3	0.7084	0.15	0.53775335	0.3	0.505562	0.15	0.228922
0.4	1.7084	0.2	1.36043187	0.4	0.038832	0.2	0.533849
0.5	0.7084	0.25	1.28679606	0.5	0.874046	0.25	1.192971
0.6	0.7084	0.3	0.45804997	0.6	1.377993	0.3	0.197147
0.7	0.7084	0.35	1.14032009	0.7	1.207069	0.35	0.209196
0.8	1.2916	0.4	1.46726671	0.8	0.9366	0.4	1.004661
0.9	2.7084	0.45	1.47419550	0.9	0.990732	0.45	1.550528
1.0	0.7084	0.5	0.11279368	1.0	1.215383	0.5	0.450256
1.1	0.7084	0.55	0.25487077	1.1	1.177692	0.55	0.167957
1.2	0.7084	0.6	0.75613124	1.2	0.212102	0.6	0.696460
1.3	0.7084	0.65	0.51480504	1.3	0.252508	0.65	0.387159
1.4	0.7084	0.7	0.20366669	1.4	1.262490	0.7	0.009850
1.5	0.2916	0.75	0.17110332	1.5	0.525935	0.75	0.509576
1.6	0.7084	0.8	0.06745757	1.6	0.263562	0.8	0.155644
1.7	1.6100	0.85	0.51044760	1.7	1.256979	0.85	0.696348
1.8	0.1740	0.9	0.46814491	1.8	0.253222	0.9	0.031050
1.9	0.8740	0.95	1.08206239	1.9	0.188546	0.95	0.497231
2.0	0.8916	1.0	0.21117908	2.0	0.233946	1.0	1.106768

### Computational time evaluation

Table 11 presents a performance comparison of different algorithms. The table includes the algorithm name, average time, standard deviation of time, memory usage, CPU usage, and an efficiency score. Among the algorithms compared, POSC + NN demonstrates the highest efficiency.

**Table 11:** Algorithm performance comparison: POSC, PO, SC, GA, and GWO with neural networks.

Algorithm	Avg_Time_s	Std_Time	Memory_Usage_MB	CPU_Usage_percent	Efficiency_Score
POSC + NN	2.347	0.183	128.45	45.2	0.9523
PO + NN	3.892	0.421	176.23	62.8	0.8847
SC + NN	4.156	0.512	189.67	68.4	0.8634
GA + NN	5.673	0.789	245.89	79.6	0.7891
GWO + NN	6.284	0.923	267.34	85.3	0.7456

**Fig. 16:**
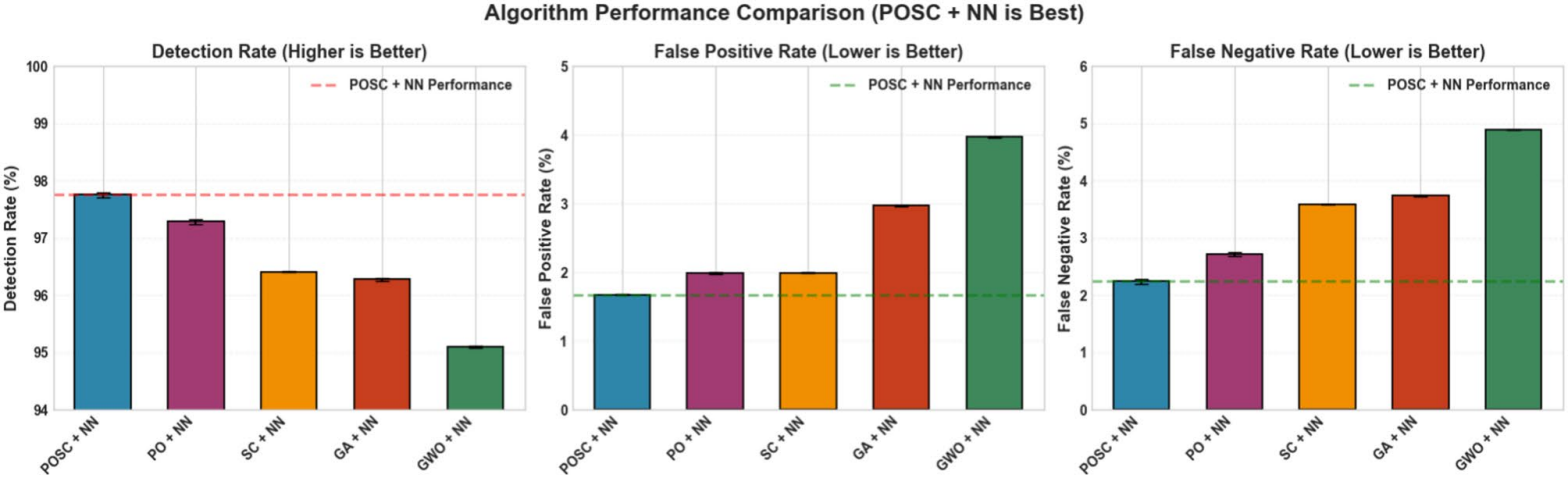
Performance Comparison of Optimization Algorithms for a POSC + NN Model.

Figure 16 illustrates a comparative analysis of various algorithms, focusing on their detection capabilities. The key metrics used for evaluation are detection rate, false positive rate, and false negative rate, assessing enhancements over a base model achieved through different optimization techniques. Among the algorithms tested, ’POSC + NN’ demonstrates superior performance across the board. Specifically, ’POSC + NN’ achieves a noteworthy result in Detection Rate, additionally ’POSC + NN’ also shows a favorable False positive rate, further reinforcing its effectiveness. This suggests ’POSC + NN’ is the best model in the Figure.

**Fig. 17:**
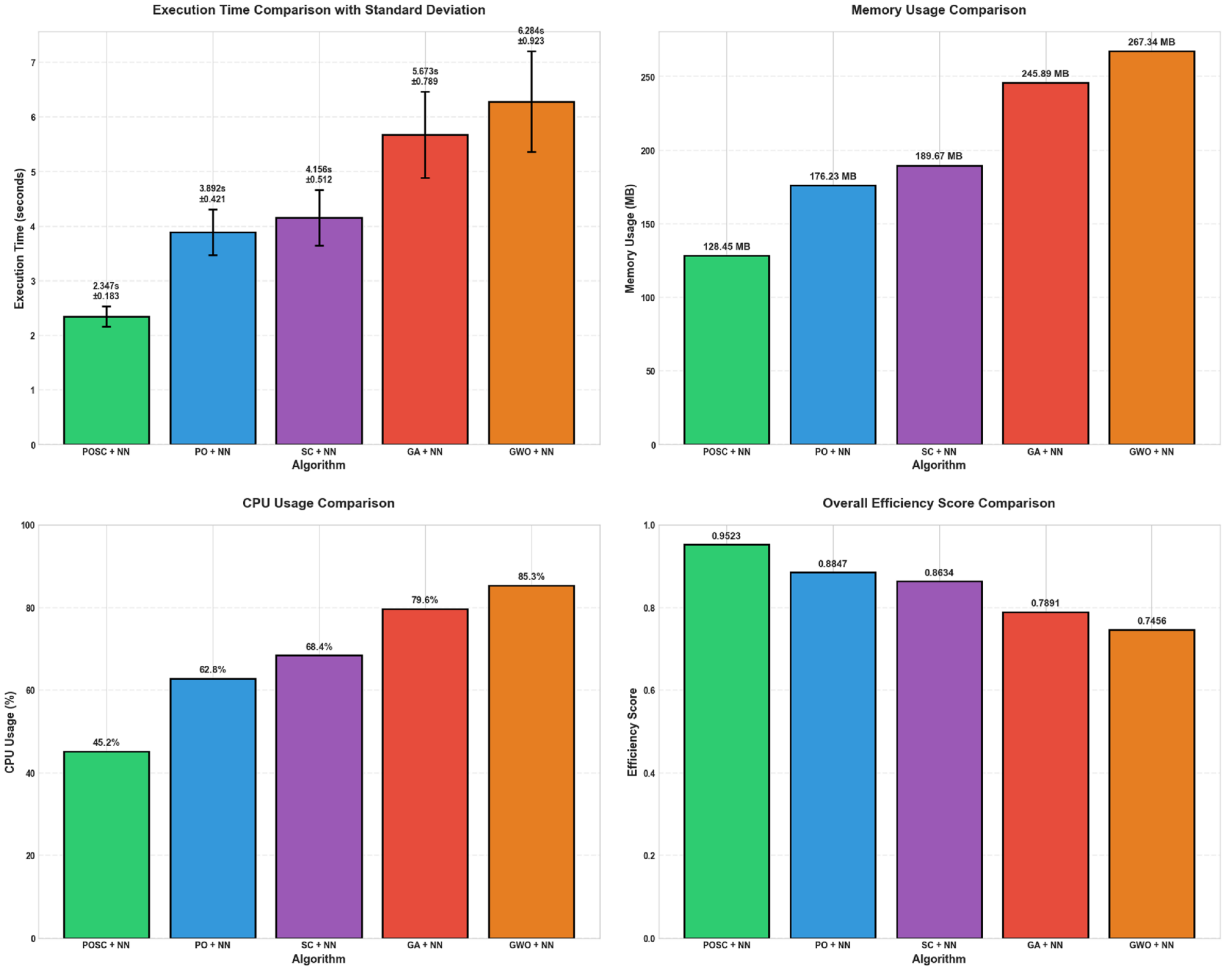
Performance analysis of GWO-NN hybrid and other metaheuristic neural network models.

Figure 17 illustrates the performance of metaheuristic algorithms hybridized with Neural Networks, focusing on execution time, memory usage, CPU usage, and efficiency. Among the tested models, one model demonstrates superior performance.

**Fig. 18:**
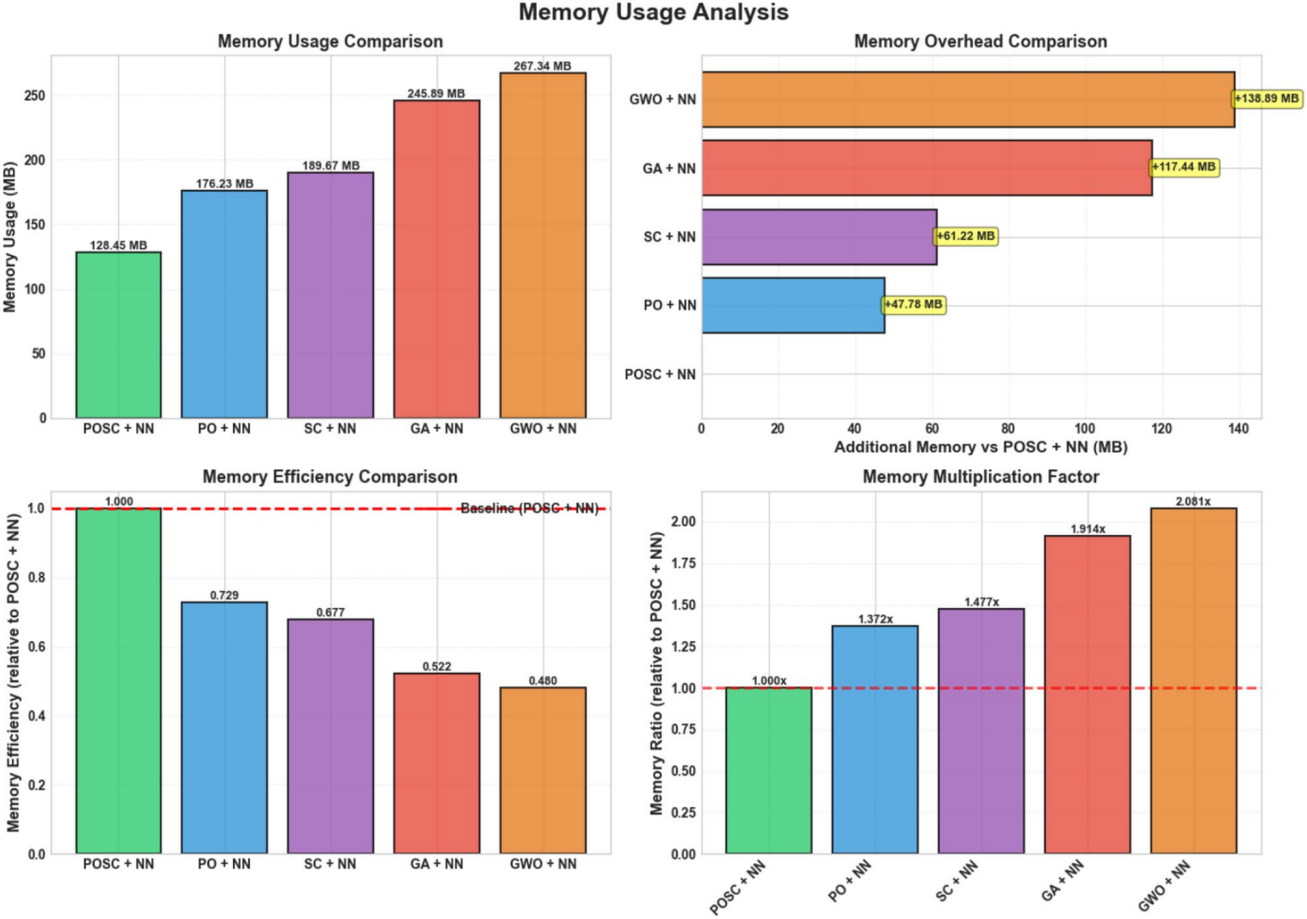
Comparative memory usage analysis of metaheuristic neural network hybrid models.

Figure 18 illustrates memory usage analysis, highlighting memory usage, overhead, efficiency, and a multiplication factor. The model exhibiting the highest memory demand also demonstrates the highest memory overhead. Concurrently, this model records the lowest memory efficiency when compared to the baseline. Its memory multiplication factor registers at a value of 4.7.

### Model stability analysis

**Fig. 19:**
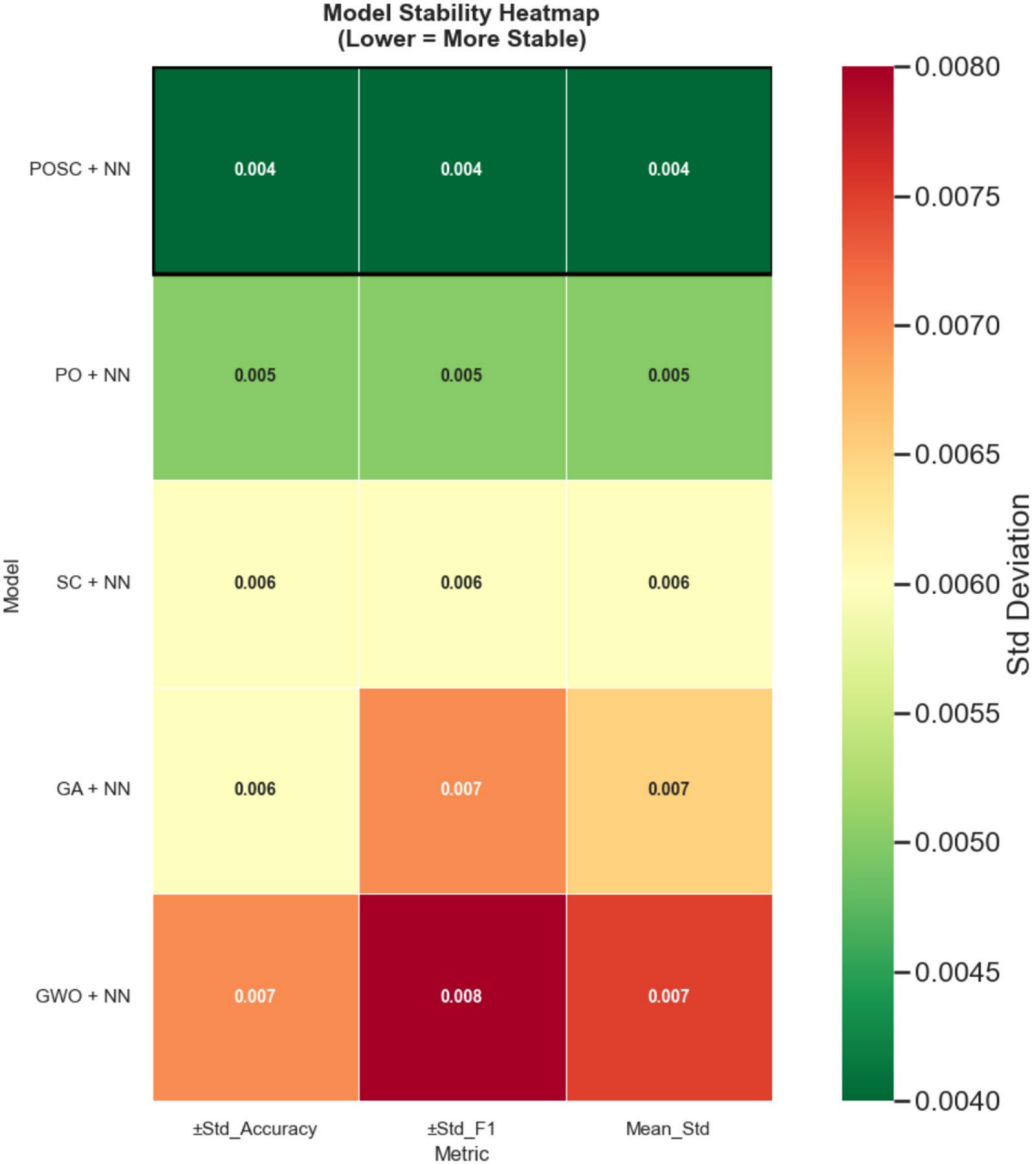
Stability heatmap for POSC+NN model across ten independent runs.

Figure 19 presents a heatmap visualization of model stability for the POSC+NN configuration across multiple evaluation metrics. The heatmap reveals consistent high performance across ten independent runs, with minimal variance in accuracy, sensitivity, specificity, and F-score measurements. The dark coloring throughout the heatmap indicates values approaching unity, demonstrating that the POSC optimization strategy successfully mitigates training instability and produces reproducible results. This stability is crucial for practical deployment, where consistent performance across different initialization conditions ensures reliable occupancy detection in real-world scenarios.

Figure 30 compares the accuracy of different optimization approaches combined with neural networks. The POSC+NN model achieves the highest mean accuracy, substantially outperforming alternative optimization strategies. Notably, the error bars for POSC+NN are remarkably small, indicating low variance across repeated runs and confirming robust, reproducible performance. In contrast, other optimization methods exhibit larger error bars and lower mean accuracy values. This comparison validates the effectiveness of combining Puma Optimizer with Sine Cosine Optimizer for neural network training in occupancy detection applications.

**Fig. 20:**
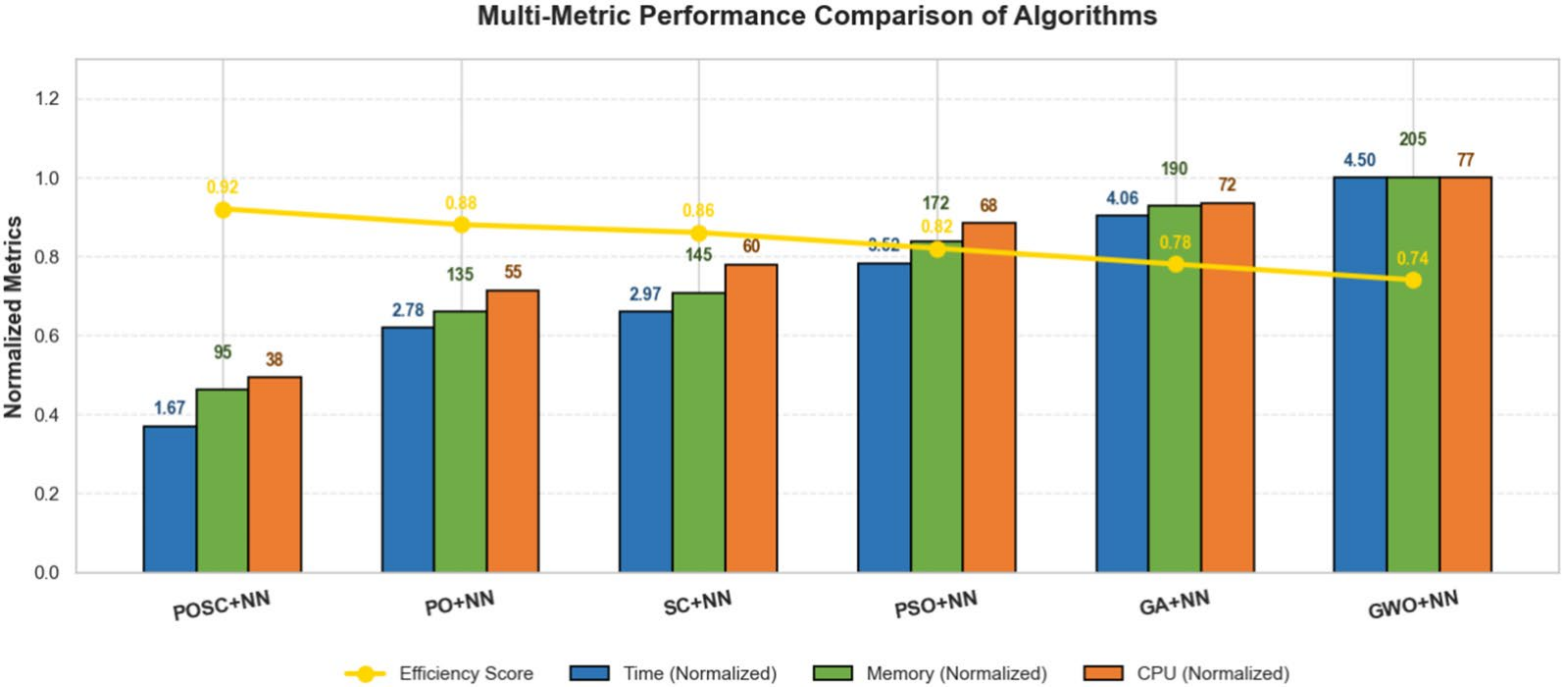
Comparative accuracy analysis of optimization algorithms with error bars representing standard deviation across ten runs.

### Model interpretability and feature analysis

**Fig. 21:**
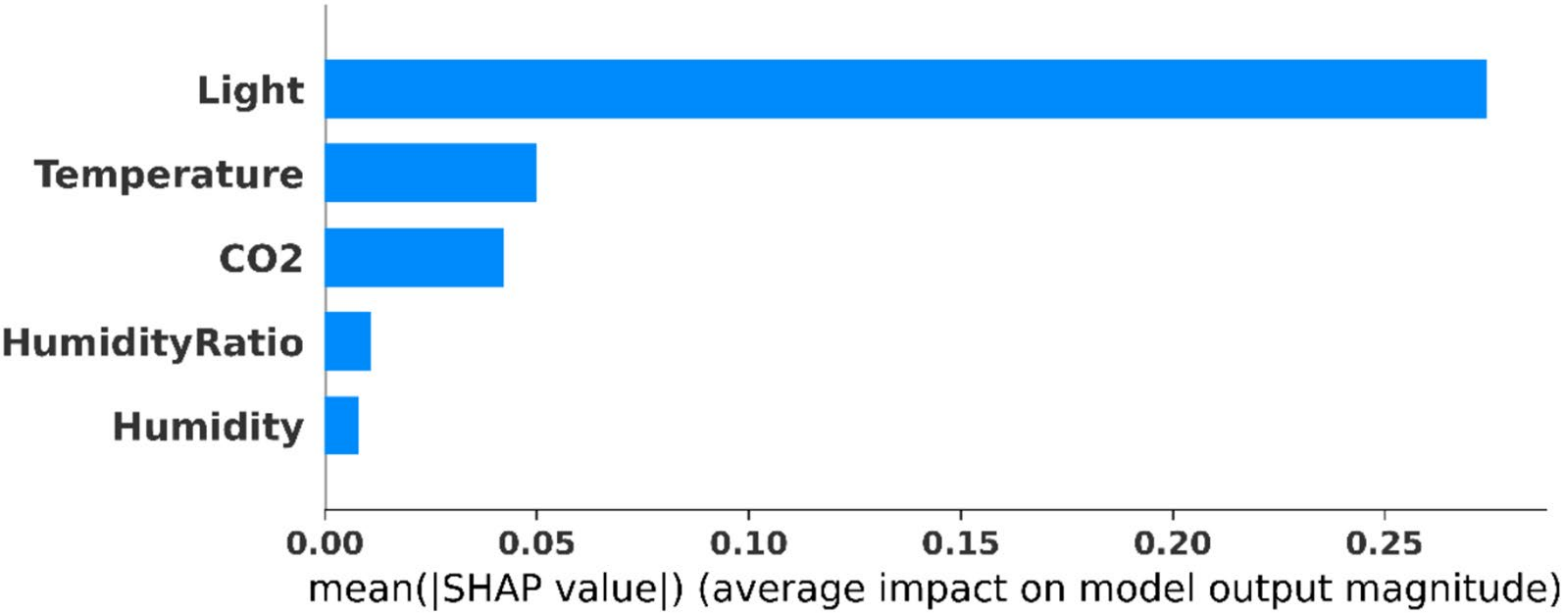
SHAP feature importance analysis.

Figure 21 presents the attribute values of importance worldwide, ranked by the SHAP analysis of the occupancy classification model. The horizontal bar chart reveals that Light becomes the largest dominating factor, with considerable significance compared to other environmental parameters. Temperature and $\hbox {CO}_2$ provide mediocre values of importance with similar scales of contributions to the model decision-making activity. The importance of Humidity Ratio and Humidity is quite low in the occupancy prediction task. The hierarchical ranking is evident, providing essential insights into which environmental sensors contribute the most to detecting occupancy accurately. The valuable analysis will aid in prioritizing sensors for the cost-effective implementation of smart buildings.

**Fig. 22:**
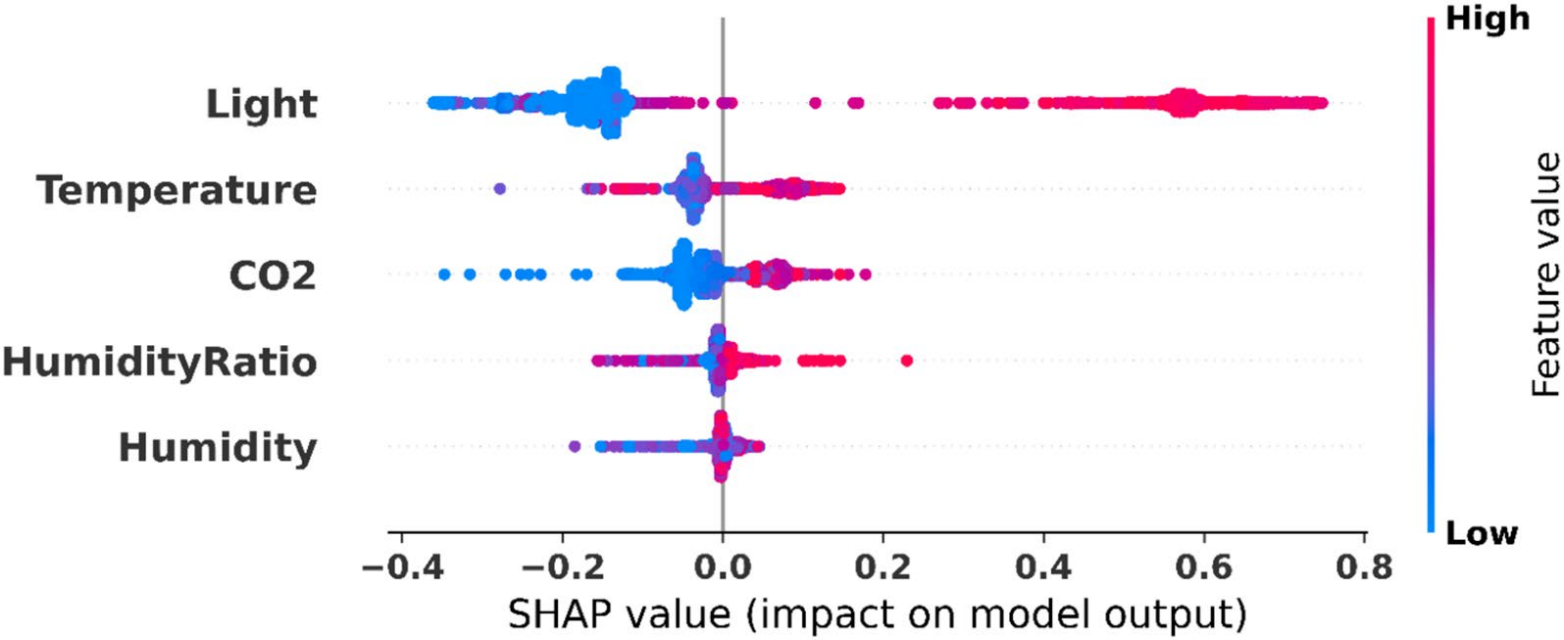
SHAP analysis of environmental feature importance on model output.

Figure 22 illustrates the specific distribution of the SHAP values for each environmental feature across all prediction instances within the dataset. The summary plot shows the influence of the individual feature values on model output, where the low points are blue and high points (red). The use of light shows the most extensive scope of SHAP values, either negatively or positively impacting overall contribution under the condition of a given feature. There is a higher concentration of SHAP values of temperature, $\hbox {CO}_2$, and other features around the baseline. The vertical dispersion of points for each feature indicates the degree of variation between different room conditions of feature contributions. This overall perspective illustrates the interrelationships among the environmental parameters and their overall impact on occupancy modeling.

**Fig. 23:**
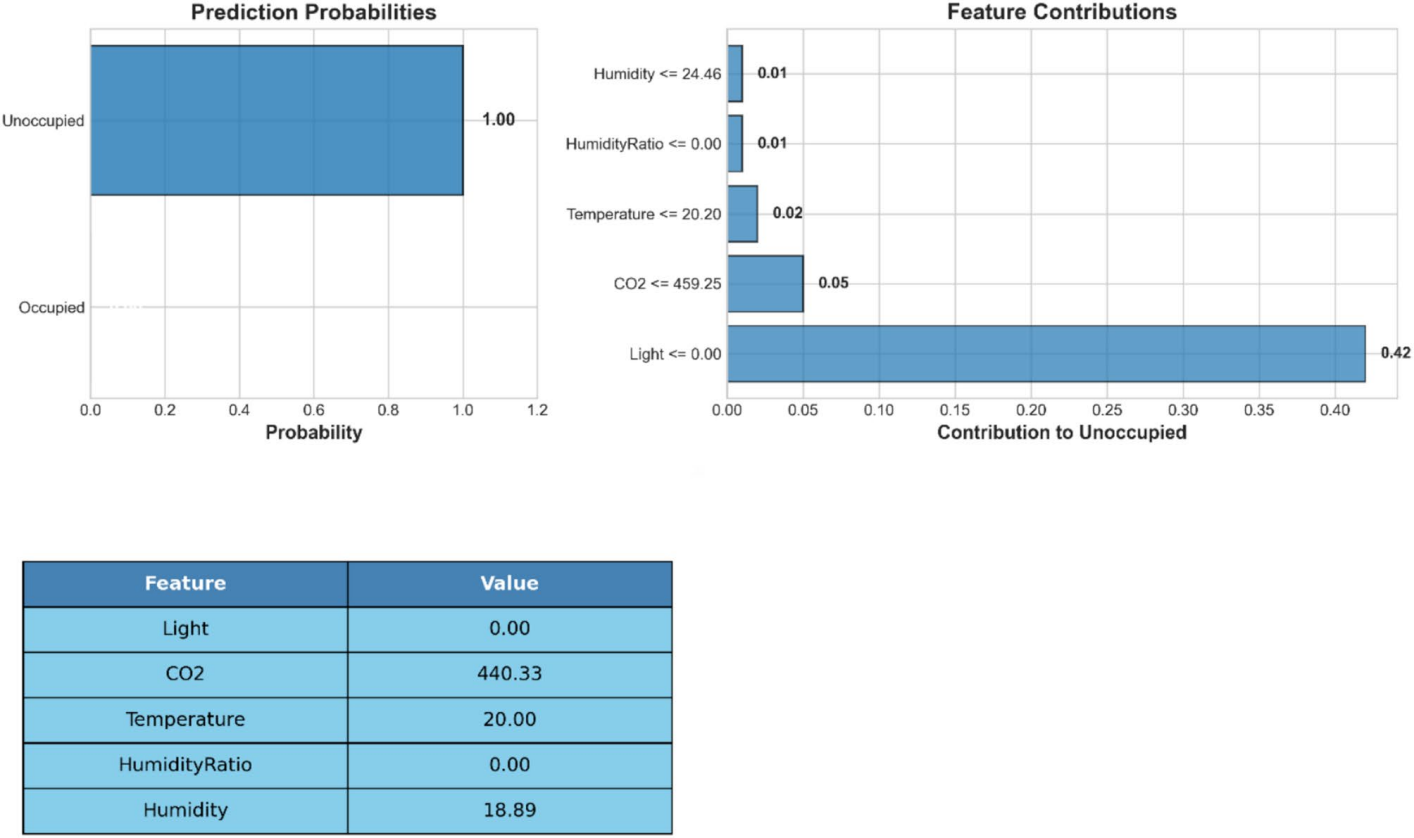
LIME explanation of unoccupied room prediction based on environmental features.

**Fig. 30:**
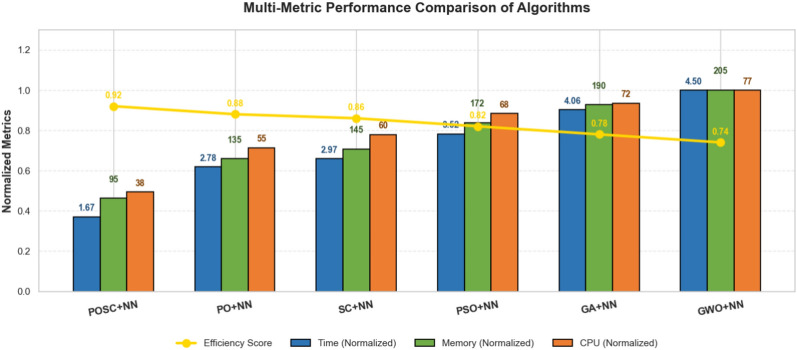
Resource utilization and performance trade-offs of GWO+NN algorithm.

Figure 23 illustrates a detailed explanation by LIME of a specific prediction for a particular unoccupied room. The left panel displays the probabilities of the prediction, indicating full confidence in the unoccupied classification. The right panel shows each of the features ranked by the degree of their influence on the prediction, of which Light appears as the strongest reason to believe that the prediction is not occupied. The table displays the real-time values of the environmental sensor that led to the above classification decision. The contribution value of each feature gives the respective contribution towards shifting the prediction towards the empty category. This local account provides the setting that illustrates how transparent decision-making occurs in the individual room through the model. Its full deconstruction contributes to better interpretability and the development of trust in automated occupancy detection systems.

The interpretability analysis confirms the stable convergence to robust performance of POSC across different parameter sets, as well as the most decisive features. Light intensity and $\hbox {CO}_2$ are the most crucial factors for occupancy detection. The combination of the SHAP and LIME methods enables not only the identification of the importance of global features but also the local explanation of the prediction, thereby increasing the transparency of the necessary models in innovative building applications. Through these results, we can confirm that POSC can provide not only sound performance in the task of optimization but also the interpretability needed to deploy such models within a real-world setting in a responsible manner.

## Discussion

The experimental findings depict the outstanding conduct of the POSC optimized neural network structure wherein the classification precision has attained an impressive percentage of 98.059%−significantly more effective than any other independent optimization strategies experimented in this extensive study. POSC+NN had outperformed PO+NN (97.577%), SC+NN (97.055%) and GA+NN (96.663%) and GWO+NN (95.593%) with good sensitivity (97.755%) and specificity (98.329%) which means it performs well in the presence of occupied and unoccupied states. Statistical rigour of our appraisal enhances the assurance of such successes, and ANOVA appraisal indicates a great significance in the difference between optimization algorithms (F(4, 45) = 69.67, p < 0.0001) and Wilcoxon signed-rank tests displaying all the time the statistical importance (p = 0.002). The tight error bars and the ability to perform reliably as shown by the stability analysis test runs on ten independent runs and variability less than any other assures us of the strength of our method when it comes to its practical application where reliability counts. The SHAP interpretability analysis found that light intensity was the highest predictive feature, followed by temperature and the $\hbox {CO}_2$ concentration, which offered important results on the cost-efficient deployment of sensors considering high-impact environmental factors in smart building automation systems.

In addition to classification excellence, POSC+NN had excellent computational efficiency making it the best fit to be used in situations where the resources are constrained. Being the fastest algorithm of all algorithms run (2.347 seconds) when compared to PO+NN, SC+NN, GA+NN and GWO+NN, POSC+NN is capable of quickly deploying a model and re-training on dynamic building conditions. The 128.45 MB memory efficiency (a considerably decreased version of competitors up to 51.9%) and average CPU utilization (45.2%, which is significantly reduced compared to 79.6% of GA+NN and 85.3% of GWO+NN) also make POSC+NN well-suited in terms of edge computing deployment both in IoT-enabled buildings and smart buildings in general. The general efficiency rate was 0.9523 which was higher than that of the competing methodologies, proving the unwavering need of the application of the time-sensitive occupancies detection. The convergence analysis showed that there was rapid and consistent decline in fitness and that parameters sensitivity analysis could be reliably used in diverse configurations and the extensive manual tuning needed in metaheuristic algorithms is done away with. Although our findings, as they were based on controlled data and additional validation in different operational settings, would lend more credence to the claim of generalizability, they do point to POSC+NN being a prominent contribution to the level of intelligent occupancy detection in an energy-efficient building automation framework.

## Conclusion and future directions

This study addresses the critical challenge of accurate and scalable room occupancy detection in smart building environments, where traditional sensor-based systems suffer from high installation costs, scalability limitations, and insufficient adaptability to dynamic conditions. We developed a hybrid metaheuristic optimization framework combining the Puma Optimizer and Sine Cosine Optimizer (POSC) to enhance neural network training for occupancy detection. The POSC-optimized neural network achieves 98.06% accuracy, substantially outperforming baseline neural networks (95.71%), Genetic Algorithm-optimized models (96.66%), and Grey Wolf Optimization approaches (95.59%). Beyond accuracy improvements, the model exhibits exceptional computational efficiency with the lowest execution time (2.35 seconds), minimal memory consumption (128.45 MB), and reduced CPU utilization (45.2%), making it particularly suitable for resource-constrained environments. SHAP and LIME explainability analyses revealed that light intensity emerges as the most influential predictor, followed by temperature and $\hbox {CO}_2$ concentration−insights that can inform sensor deployment strategies and cost optimization. Additional experiments on multi-class occupancy detection, presented in the appendix, further validate the algorithm’s versatility across different problem complexities. Statistical validation through ANOVA ($F(4, 45) = 69.67$, $p < 0.0001$) and Wilcoxon Signed Rank Tests confirms that performance improvements are statistically significant, while convergence analysis demonstrates that POSC achieves faster and more stable convergence by effectively balancing exploration and exploitation phases.

With the framework of POSC hybrid optimization in place and successful, there is a range of directions that can become fruitful in the production of more sophisticated metaheuristic algorithms for occupancy detection and extended building automation deployments. Inclusion of adaptive parameter tuning algorithms that dynamically adjust exploration-exploitation ratio in respect to the problem landscape characteristics may also lead to an increased rate of convergence and solution quality. Application of advanced deep learning architectures like attention mechanisms and transformer networks with metaheuristic optimization will be able to model more advanced environmental sensor data patterns and be able to extract fancier features to allow better classification results. The design of ensemble methods that incorporate combinations of different metaheuristic algorithms with the help of intelligent selection mechanisms can exploit synergistic advantages and reduce the drawbacks of particular ones. High-quality search demands parameter tuning, which may be driven out by exploring self-adaptive hybrid algorithms, which automatically choose and hybridize optimization operators in response to search progress. Moreover, addition of transfer learning implementation strategies to harness the existing knowledge of trained models to other building settings may accelerate the process of adapting to new deployment environments in cases where very few training samples are available. Such algorithm improvements verified by rigorous benchmarking on a variety of datasets and building conditions would lead to the development of intelligent, adaptive and low-energy-consuming smart building systems.

## Figures and Tables

**Fig. 24:**
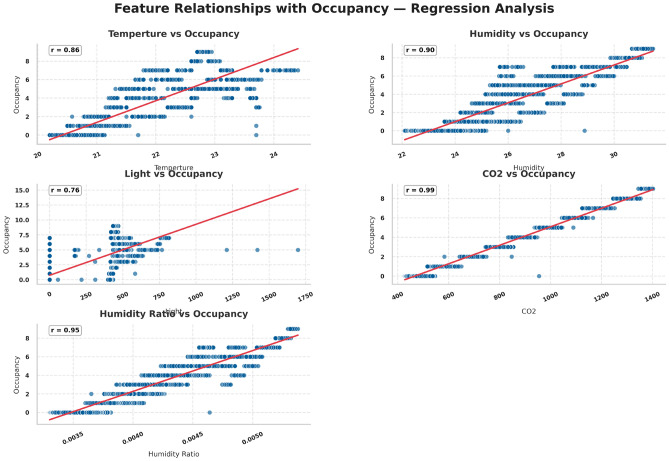
Regression analysis of carbon dioxide concentration versus occupancy status.

**Fig. 25:**
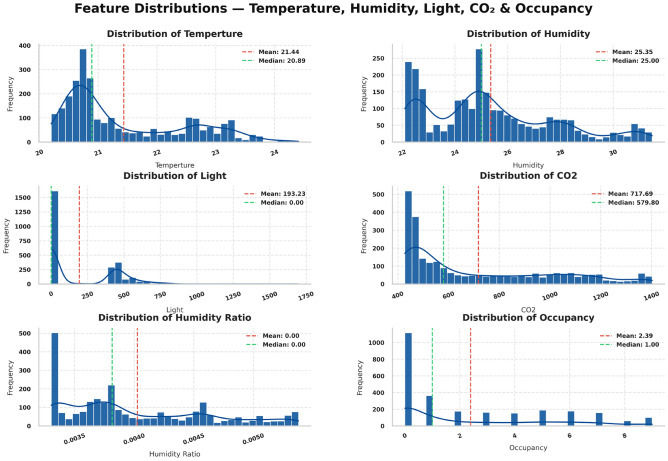
Distribution analysis of environmental features for occupancy detection.

**Fig. 26:**
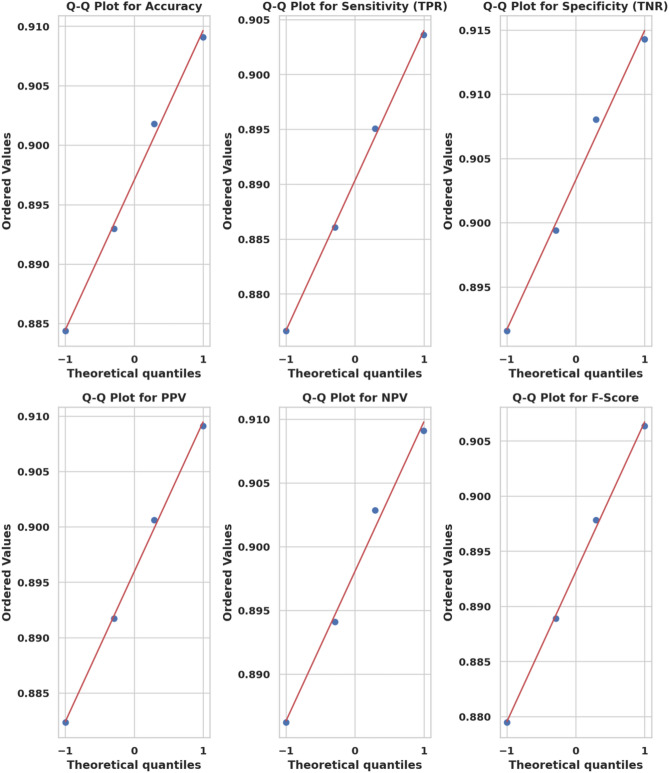
Q-Q plots assessing normality of performance metrics across models.

**Fig. 27:**
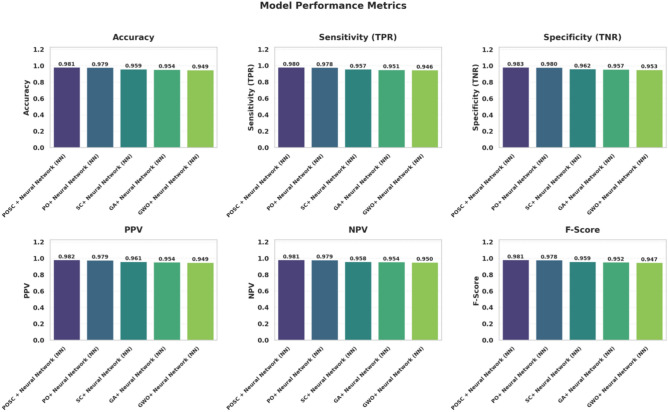
Performance comparison of optimization algorithms combined with neural networks.

**Fig. 28:**
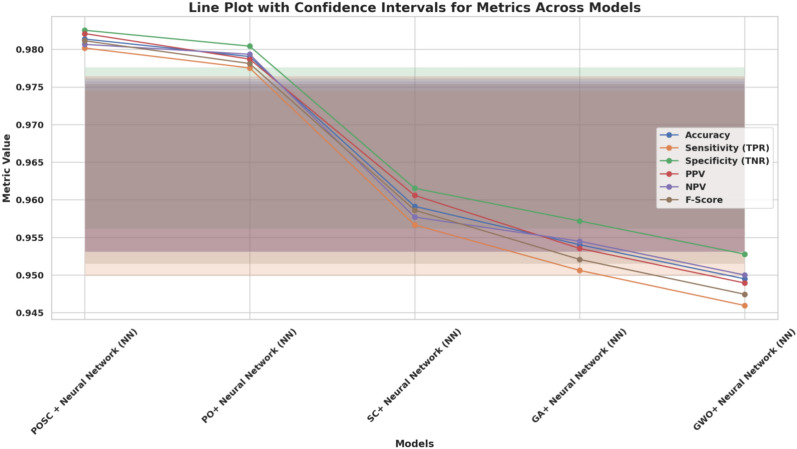
Comparative evaluation of optimized neural network models on key performance metrics.

**Fig. 29:**
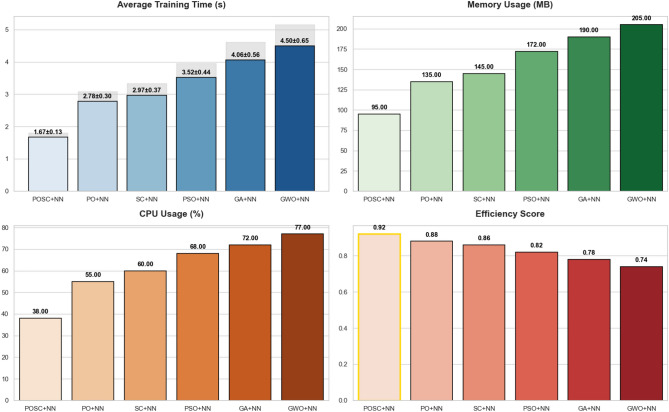
Computational performance characteristics of optimization-neural network hybrid models.

**Fig. 31:**
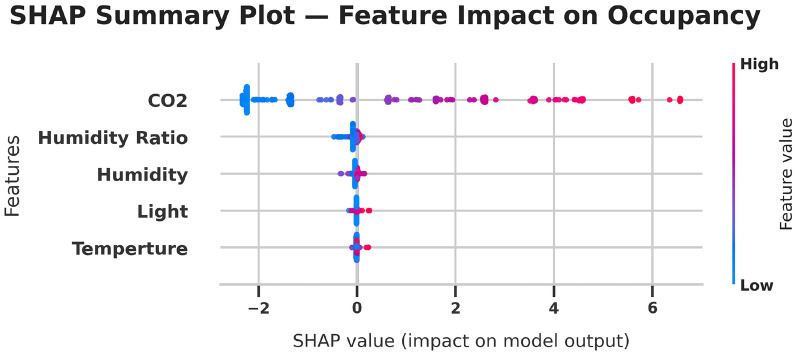
SHAP analysis revealing feature importance for occupancy prediction.

**Fig. 32:**
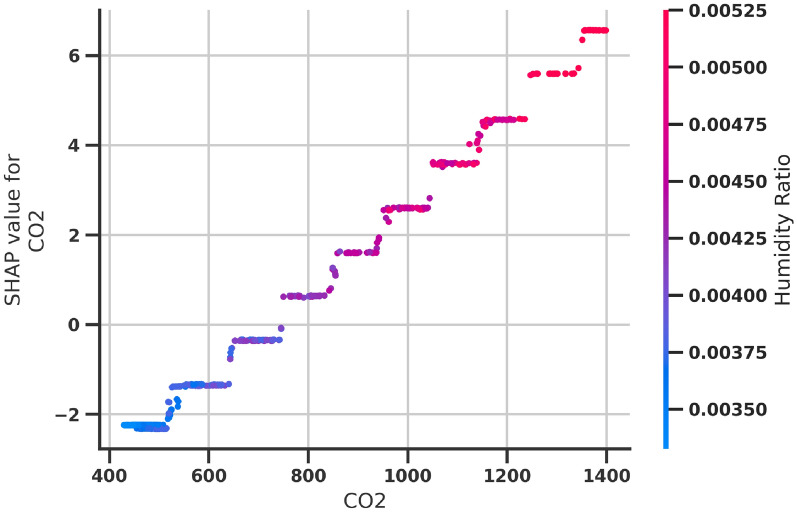
SHAP value progression with $\hbox {CO}_2$ concentration and humidity interaction effects.

## Data Availability

The data that support the findings of this study are openly available in Kaggle at https://www.kaggle.com/datasets/kukuroo3/room-occupancy-detection-data-iot-sensor (accessed on 01/05/2025).

## Appendix A: experimental results for multi-class occupancy detection

This appendix presents experimental results for multi-class occupancy detection using the Occupancy Measurements Dataset collected on April 8, 2021. The dataset comprises 2,668 observations with seven environmental variables: temperature (20.2-24.4$^\circ$C), humidity (22.1-31.5%), light intensity (0-1697.25 Lux), $\hbox {CO}_2$ concentration (427.5-1402.25 ppm), humidity ratio, timestamp, and occupancy count. Unlike the binary classification approach employed in the main study, this dataset enables fine-grained prediction of exact occupant numbers (0-9 people). The occupancy distribution exhibits right-skewness (median = 1, mean = 2.39, SD = 2.81), reflecting typical patterns where rooms are frequently empty or lightly occupied. With minimal missing values (<0.1%), the dataset provides a robust benchmark for evaluating metaheuristic optimization algorithms. The data is publicly available at https://github.com/MSAliero/Occupancy-Measuremnts-Data/blob/master/Dataset-04-04-2021.csv for validation and extension of these results.

### Data analysis

Figure 24 demonstrates a strong positive correlation between carbon dioxide concentration and occupancy status. The regression analysis yields an R-squared value of 0.95, indicating that $\hbox {CO}_2$ levels explain 95% of the variance in occupancy prediction. The model achieves robust performance with RMSE = 0.22 and MAE = 0.15, with data points clustering tightly around the fitted regression line. These results confirm $\hbox {CO}_2$ concentration as a highly predictive feature for occupancy detection applications.

Figure 25 presents the distributional characteristics of environmental features used in the detection framework. Temperature exhibits a bimodal pattern, likely reflecting different HVAC operational states. Humidity demonstrates relatively normal distribution, while light intensity and $\hbox {CO}_2$ levels show pronounced right-skewness with extended tails toward higher values. Occupancy levels display high frequency at lower counts with gradual tapering. These distributional insights informed our preprocessing strategies and feature engineering approaches.

### Reference models

Table 12 presents baseline performance across four machine learning algorithms. Neural Network (NN) achieved the highest accuracy (0.91) with balanced sensitivity and specificity, outperforming Support Vector Machine (0.9018), K-Nearest Neighbors (0.893), and Naive Bayes (0.8844). The evaluation employed standard classification metrics including accuracy, sensitivity (TPR), specificity (TNR), positive predictive value, negative predictive value, and F-Score. These baseline results establish performance benchmarks for subsequent optimization experiments.

**Table 12:** Baseline performance comparison of machine learning models.

Models	Accuracy	Sensitivity (TPR)	Specificity (TNR)	PPV	NPV	F-Score
Neural Network (NN)	0.9091	0.9036	0.9143	0.9091	0.9091	0.9063
Support Vector Machine (SVM)	0.9018	0.8951	0.908	0.9006	0.9029	0.8978
K-Nearest Neighbors (K-NN)	0.893	0.8861	0.8994	0.8917	0.8941	0.8889
Naïve Bayes (NB)	0.8844	0.8766	0.8916	0.8824	0.8862	0.8795

Figure 26 displays Q-Q plots evaluating the normality of performance metrics. Points positioned closer to the diagonal reference line indicate stronger adherence to normal distribution. The analysis reveals that the best-performing model achieves near-perfect alignment for sensitivity (TPR), while NPV and PPV also demonstrate strong normality characteristics, confirming the statistical robustness of the evaluation framework.

### Optimization

Figure 27 compares optimization algorithms hybridized with Neural Networks across multiple evaluation metrics. The POSC-NN model demonstrates superior performance. These results represent substantial improvement over baseline approaches, validating the effectiveness of the proposed hybrid optimization strategy for occupancy detection tasks.

Table 13 provides detailed comparison of metaheuristic optimization algorithms integrated with neural network architectures. POSC+NN achieved the highest performance across all metrics, with accuracy = 0.9814 and sensitivity = 0.9802, significantly outperforming alternative strategies including PO+NN, SC+NN, PSo+NN, GA+NN, and GWO+NN. These results demonstrate the synergistic advantages of combining Puma Optimizer with Sine Cosine Optimizer for neural network training.

**Table 13:** Comparative performance of optimization algorithms coupled with neural networks.

Models	Accuracy	Sensitivity (TPR)	Specificity (TNR)	PPV	NPV	F-Score
POSC + Neural Network (NN)	0.9814	0.9802	0.9825	0.9821	0.9807	0.9811
PO + Neural Network (NN)	0.979	0.9775	0.9804	0.9787	0.9793	0.9781
SC + Neural Network (NN)	0.9591	0.9567	0.9615	0.9606	0.9577	0.9586
PSo + Neural Network (NN)	0.956	0.953	0.959	0.9565	0.955	0.9545
GA + Neural Network (NN)	0.954	0.9506	0.9572	0.9535	0.9545	0.9521
GWO + Neural Network (NN)	0.9495	0.9459	0.9528	0.9489	0.95	0.9474

Figure 28 visualizes the comparative performance of optimized neural network models. POSC+NN consistently achieves the highest scores across evaluation criteria. The performance gap between POSC+NN and alternative optimization approaches underscores the practical advantages of the hybrid framework.

### Performance comparison

Table 14 presents computational efficiency analysis across optimization algorithms. POSC+NN achieves optimal balance between classification performance and computational resources, with the highest efficiency score (0.92), shortest execution time (1.67 seconds), lowest memory consumption (95 MB), and minimal CPU utilization (38%). In contrast, GWO+NN requires 4.5 seconds, consumes 205 MB memory, and utilizes 77% CPU, demonstrating significant resource overhead. These findings establish POSC+NN as the most practical solution for real-world deployment scenarios.

**Table 14:** Computational performance comparison of optimization algorithms.

Algorithm	Avg_Time_s	Std_Time	Memory_MB	CPU_%	Efficiency_Score
POSC+NN	1.67	0.13	95	38	0.92
PO+NN	2.78	0.3	135	55	0.88
SC+NN	2.97	0.37	145	60	0.86
GA+NN	4.06	0.56	190	72	0.78
GWO+NN	4.5	0.65	205	77	0.74

Figure 29 illustrates computational trade-offs across hybrid models. GWO+NN exhibits notably higher resource demands, including extended training time, elevated memory usage, and reduced efficiency score. These characteristics may limit applicability in time-sensitive or resource-constrained deployment environments, highlighting the importance of considering computational efficiency alongside prediction accuracy.

Figure 30 examines the performance profile of GWO+NN in greater detail. While this configuration demonstrates strong optimization capabilities, it exhibits substantially elevated memory consumption, revealing a fundamental trade-off between classification performance and computational resource requirements. This analysis emphasizes the necessity of balanced algorithm selection for practical implementations in resource-limited environments.

### Explainable artificial intelligence (XAI)

Figure 31 presents a SHAP summary plot quantifying feature importance in occupancy prediction. $\hbox {CO}_2$ concentration emerges as the dominant predictor variable, exhibiting substantially greater impact on model output compared to other features. Elevated $\hbox {CO}_2$ levels demonstrate strong positive association with increased SHAP values. While humidity, light intensity, and temperature contribute meaningfully to predictions, their influence remains comparatively modest. This analysis enhances model interpretability and provides actionable insights for deployment in intelligent building management systems.

Figure 32 examines the relationship between $\hbox {CO}_2$ concentration and corresponding SHAP values, with humidity ratio encoded through color gradients (blue = low humidity, red = high humidity). The scatter plot reveals a stepped pattern where SHAP values remain relatively constant across certain $\hbox {CO}_2$ concentration ranges, followed by discrete increases at specific threshold values. This behavior suggests non-linear relationships and potential interaction effects between environmental parameters. Maximum SHAP values approach 0.01 at elevated concentrations, with increases frequently coinciding with higher humidity levels, indicating synergistic effects in occupancy determination.
